# Database of Shear Experiments on Steel Fiber Reinforced Concrete Beams without Stirrups

**DOI:** 10.3390/ma12060917

**Published:** 2019-03-19

**Authors:** Eva O. L. Lantsoght

**Affiliations:** 1 Politécnico, Universidad San Francisco de Quito, Quito 170901, Ecuador; elantsoght@usfq.edu.ec or e.o.l.lantsoght@tudelft.nl; Tel.: +593-2-297-1700 (ext. 1186); 2 Concrete Structures, Department of Engineering Structures, Civil Engineering and Geosciences, Delft University of Technology, 2628 CN Delft, The Netherlands

**Keywords:** beams, database, experiments, flexure, shear, steel fiber reinforced concrete

## Abstract

Adding steel fibers to concrete improves the capacity in tension-driven failure modes. An example is the shear capacity in steel fiber reinforced concrete (SFRC) beams with longitudinal reinforcement and without shear reinforcement. Since no mechanical models exist that can fully describe the behavior of SFRC beams without shear reinforcement failing in shear, a number of empirical equations have been suggested in the past. This paper compiles the existing empirical equations and code provisions for the prediction of the shear capacity of SFRC beams failing in shear as well as a database of 488 experiments reported in the literature. The experimental shear capacities from the database are then compared to the prediction equations. This comparison shows a large scatter on the ratio of experimental to predicted values. The practice of defining the tensile strength of SFRC based on different experiments internationally makes the comparison difficult. For design purposes, the code prediction methods based on the Eurocode shear expression provide reasonable results (with coefficients of variation on the ratio tested/predicted shear capacities of 27–29%). None of the currently available methods properly describe the behavior of SFRC beams failing in shear. As such, this work shows the need for studies that address the different shear-carrying mechanisms in SFRC and its crack kinematics.

## 1. Introduction

When steel fibers are added to the concrete mix, the weak tension properties of the concrete may be improved, as the steel fibers can carry this tension. As a result, steel fiber reinforced concrete (SFRC) has superior material and mechanical behavior for all tension-driven material properties and failure modes. An example of a tension-driven failure mode is shear failure [1,2]. Typically, shear-critical elements are provided with shear reinforcement. However, for certain cases, providing shear reinforcement may not be desirable. One example of such an application is reinforced concrete one-way slabs [3], where using shear reinforcement is often not cost-effective. For other cases, heavy shear reinforcement and the resulting reinforcement congestion make casting concrete difficult [4], especially in high performance high strength beams, so that other solutions may be more practical and may lead to a better execution and performance of the structural element. For these cases, dispersing steel fibers in the concrete mix can improve the shear capacity and reduce or eliminate the need for stirrups.

Bernard proposed the use of steel “splinters” to strengthen concrete in tension as early as 1874 [5]. Nevertheless, practical applications of SFRC are still not widespread. The main barrier to application is that building codes, such as the ACI 318-14 [6] and EN 1992-1-1:2004 [7] do not contain provisions for determining the shear capacity of SFRC. The most noteworthy national codes and guidelines with shear provisions for SFRC are the French recommendations [8,9,10], the German guideline [11], and the Italian guide [12].

The currently available shear equations from codes and guidelines, as well as those reported in the literature are summarized in this work. An analysis of the available expressions shows that the majority are empirical equations. Expressions resulting from an analysis of the mechanics of the problem are scarce, with the exceptions of the extensions [13,14] of the modified compression field theory (MCFT) [15] and the dual potential capacity model [16,17]. None of the existing expressions are based on an analysis of the shear-carrying mechanisms in concrete structures [18]: the capacity of the uncracked concrete in the compression zone [19], aggregate interlock [20], dowel action [21], and residual tension across the crack [22]. For SFRC, the contribution of the residual tension across the crack may be negligible, and instead the contribution of the steel fibers bridging the crack should be analyzed [23]. This lack of understanding of the mechanics of the problem forms a more fundamental barrier to the practical application of SFRC. To optimize structural designs, and reduce the material quantities used in a project, as well as their embodied carbon and environmental impact, it is important to develop better models for the shear capacity of SFRC with longitudinal steel reinforcement without stirrups.

Before better models for the shear capacity of SFRC can be evaluated, it is necessary to gather the available experimental data from the literature. This information can be used to analyze the shortcomings of the current equations, and to carry out parameter studies. This paper presents a unique database of 488 experiments. Smaller databases have been reported or discussed in the literature previously [24,25,26,27,28,29,30], but the current effort has resulted in the gathering of a significantly larger number of datapoints. Moreover, the full database is available as a dataset in the public domain for other researchers [31], which is a step forward as well.

## 2. Methods

### 2.1. Overview of Shear Prediction Equations

The currently available expressions to predict the shear capacity of SFRC beams without stirrups are mostly empirical equations. Besides the empirical equations, some methods have been derived that are (partially) based on the mechanics of the problem. Noteworthy here are extensions of the modified compression field theory (MCFT) [15,32] for SFRC, the dual potential capacity model [16,17], and plasticity-based approaches. For the extension of the MCFT to SFRC several approaches have been followed: describing the constitutive equations of cracked SFRC [33,34], assumptions for smeared cracking in SFRC [35], programming the effect of fibers into the VecTor2 software [13,36], panel testing [37], the development of an engineering model [38] for inclusion in the next version of the *fib* model code [39], and the development of a model that considers the rotation of the individual fibers with respect to the crack plane [40] and its closed-form solution [41]. Hwang’s softened truss model with steel fibers [42] falls in the same category as the MCFT for SFRC. Most of these MCFT-based methods require programming and/or the use of finite element models. The dual potential capacity model [16,17] evaluates the capacity of the concrete in the compression zone and the tension capacity of the SFRC in the tension zone. The drawback of this approach is that these assumptions for the mechanics of the behavior do not reflect all shear-carrying mechanisms in SFRC (capacity of compression zone, dowel action, tension capacity of SFRC in tension zone, aggregate interlock, and arching action [18]). Plasticity-based models have been proposed in the past [43,44,45,46]. While the results of these models seem promising, they require further research and validation.

Since the mechanical models that are available in the literature each have drawbacks as pointed out in the previous paragraph, the current codes and guidelines are based on empirical models. Therefore, in this section, an overview of a selection of currently available empirical prediction equations and code equations is given. These prediction models will be used in Section 3 for comparison to the shear capacities obtained from the literature.

Table 1 gives an overview of the shear prediction equations. All symbols used in Table 1 can be found in the list of notations at the end. The expression by Sarveghadi et al. [28] is a simplification of a matrix-based expression resulting from an analysis testing different artificial neural networks. Many expressions describe the steel fiber properties with the fiber factor *F*. The fiber factor [47] is a metric used for defining the properties of the fibers, taking into account the fiber volume fraction *V_f_*, the aspect ratio *l_f_*/*d_f_*, and the bond properties of the fiber *ρ_f_*:

(1) $$F=V_{f}\frac{l_{f}}{d_{f}}\rho_{f}$$

The expression of Kwak et al. [48] follows the form of Zsutty’s empirical equation for the shear capacity of reinforced concrete beams [49], with *v_b_* as given in Equation (4). The Greenough and Nehdi expression [50], which is a simplification of an expression resulting from genetic programming, uses a % for *ρ* instead of the actual reinforcement ratio.

Khuntia et al.’s expression [51] is a proposal to include the effect of fibers on the expression for the shear capacity of ACI 318-14 [6]. Similarly, Sharma’s proposal [52] follows the format of the ACI 318-14 code expression, and links the tensile and compressive strength of concrete through the expression by Wright [53]. Mansur et al. [54] also propose an extension of the ACI 318-14 code expression, using *σ_tu_* as recommended by Swamy and Al-Ta’an [55], which uses the fiber length correction factor *η_l_* from Cox [56], the fiber spacing from Swamy et al. [57], and the bond stress *τ* proposed by Swamy and Mangat [58]. Ashour et al. [59] propose two (sets of) equations: the first equation, Equation (17) is a proposal for extension of the ACI 318-14 [6] expressions, whereas Equations (18) and (19) are based on Zsutty’s equation [49]. Arslan’s equations [60] are also based on Zsutty’s equation [49], with the addition of the determination of the height of the compression zone *c* as proposed by Zararis and Papadakis [61]. However, this method for determining *c* ignores the contribution of the fibers on the horizontal and moment equilibrium of the cross-section.

The shear capacity equation from Bažant and Kim [62], derived from fracture mechanics of quasi-brittle materials, was extended to include the contribution of fibers by Imam et al. [63] as well as Yakoub [64] (first set of equations, Equations (25) through (27)). The second set of equations by Yakoub [64], Equations (28) through (32) is a proposal to include the effect of fibers in the shear expressions from the Canadian code CSA A23.3-04 [65], which is based on the MCFT [15].

The next entries in Table 1 are expressions from codes and guidelines. The expressions from the French recommendations [10] separate the concrete contribution to the shear-carrying capacity from the contribution of the fibers. The determination of the contribution of the fibers requires experimental data of the SFRC mix, as shown in Equations (35) through (38). An additional material safety factor *γ_E_* is added so that *γ_cf_γ_E_* = 1.5. The angle of the compression strut *θ* ≥ 30°. The value of *K* in Equation (36) can be approximated as *K* = 1.25, except when *b_w_* and *h* are less than 5*l_f_*, or the value of *K* can be determined from tension tests on the SFRC mix.

The expressions from the German guideline [11] and RILEM [66] are based on the Eurocode EN 1992-1-1:2004 [7] equations, by adding a term to represent the contribution of the steel fibers. The expressions from the *fib* Model Code [39] are based on EN 1992-1-1:2004 [7], but incorporate the effect of the fibers into the original expression. The Italian guide [12] uses the same expressions as the *fib* Model Code [39], and includes a lower bound for the shear capacity *V_min_*. In the German National Annex of the Eurocode 2, *C_Rd,c_* = 0.15, and this value is used in Equation (41) as well. The following factors are used: *γ_c_* = 1.5, $\gamma_{ct}^{f}$ = 1.25, $\alpha_{c}^{f}$ = 0.85 to account for long-term effects, and $k_{F}^{f}$ = 0.5 for shear. For cross-sections subjected to axial loads, the contribution of the steel fibers cannot be taken into account, as more experimental results are necessary to derive suitable expressions [24]. In the Italian guideline [12], the influence of axial loads is considered in the same way as in EN 1992-1-1:2004 [7]. Since this work deals with elements without axial loads, the formulas have been simplified accordingly. The expressions from the German guideline [11], RILEM [66], the *fib* Model Code [39], and the Italian guide [12] are valid for *ρ* ≤ 2%. For the *fib* Model Code expressions, *C_Rd,c_* = 0.18 and *γ_c_* = 1.5. All notations used in Table 1 are explained in the “List of notations”.

### 2.2. Database of Experiments

#### 2.2.1. Development of Database

The database developed for this study contains 488 experiments of SFRC beams with longitudinal tension reinforcement (mild steel only) and without transverse shear reinforcement failing in shear reported in the literature. The consulted references are: Singh and Jain [4], Sahoo and Sharma [67], Shoaib, Lubell, and Bindiganavile [68] (lightweight beams), Manju, Sathya and Sylviya [69], Arslan, Keskin, and Ulusoy [70], Parra-Montesinos et al. [71], Rosenbusch and Teutsch [72], Sahoo, Bhagat, and Reddy [73] (T-beams), Amin and Foster [74], Tahenni et al. [75], Narayanan and Darwish [76], Cucchiara, La Mendola, and Papia [77], Kwak et al. [48], Lim and Oh [78], Dinh, Parra-Montesinos and Wight [79], Lima Araujo et al. [80], Casanova, Rossi, and Schaller [81], Aoude et al. [82], Minelli and Plizzari [83], Kang et al. [84], Casanova and Rossi [85], Lim, Paramasivam, and Lee [44], Mansur, Ong, and Paramasivam [54], Zarrinpour and Chao [86], Noghabai [87], Randl, Mészöly, and Harsányi [88], Ashour, Hasanain, and Wafa [59], Tan, Murugappan, and Paramasivam [89], Pansuk et al. [90], Kim et al. [91], Sharma [52], Narayanan and Darwish [92], Li, Ward, and Hamza [93], Swamy, Jones, and Chiam [94], Cho and Kim [95], Greenough and Nehdi [50], Kang et al. [96], Dupont and Vandewalle [97] with further information in [98], Swamy and Bahia [99], Batson, Jenkins, and Spatney [100], Zhao et al. [101], Jindal [102], Shin, Oh, and Ghosh [103], Imam, Vandewalle, and Mortelmans [104,105], Huang, Zhang, and Guan [106], Kwak, Suh, and Hsu [107], Roberts and Ho [108], Hwang et al. [109], Spinella, Colajanni, and La Mendola [110], Chalioris and Sfiri [111], Cohen and Aoude [112], Aoude and Cohen [113], Qissab and Salman [114], Furlan and de Hanai [115], Dancygier and Savir [116], Krassowska and Kosior-Kazberuk [117], Yoo and Yang [118], Gali and Subramaniam [119], Zamanzadeh, Lourenco, and Barros [120], Shoaib, Lubell, and Bindiganaville [121], Shoaib [122], Bae, Choi, and Choi [123], and Abdul-Zaher et al. [124]. The database does not include the Keskin et al. [125] experiments, since for these specimens carbon fiber reinforced polymer (CFRP) bars were used as longitudinal reinforcement. The experiments by Khan [126] are excluded, as these specimens are subjected to a combination of shear, bending moment, and torsional moment.

Table A1 gives the database developed for this study. The full spreadsheet is available as supplementary file in .xlsx format available in the public domain [31]. The notations used in this database are given in the “List of notations”. For a number of references [42,44,50,52,54,59,67,69,70,71,72,73,75,76,77,78,80,81,83,84,85,88,89,94,96,97,98,99,100,102,103,104,106,107,109,110,111,112,115,116,117,118,119,123,124] information about the geometry of the support and loading plate was missing. These values were then approximated based on figures of the test setup in the original reference. For rollers, the contact surface was assumed to be 10 mm wide. Most specimens are rectangular beams, but the specimens in [73,81,94,99] are T-beams, in [89,90] I-beams, and in [114] non-prismatic beams. Almost all experiments are on simply supported beams in three- or four-point bending, with exception of the two-span beams in [117] and the special setup by [127] for short spans that does not allow for the development of arching action.

In terms of geometry, references [54,69,76] do not report the total length of the beam specimen. Reference [121] only reports the total length for the largest specimens. For the database entries, a similar overhang is used for the smaller specimens. Reference [54] does not report the span length, but the span and total length are estimated from the technical drawings in the original reference. The total length for the beams in [52,89,97,102] was also estimated based on the technical drawings in the paper. A practical value of overhang over the support is assumed for these cases. The results in [103] are inconsistent: the relation between the maximum load in the figures and the shear stress in the reported table is not clear. The cause of this inconsistency seems to be that the authors did not show the length correctly: the sketched span length *l_span_* appears to be the total length *l_tot_*. This correction is included in the database. References [69,81,115] do not report the effective depth. For the database entries, these values are then calculated back from the *a*/*d* ratio, or based on the rebar diameter and a 10 mm cover, as typically used in laboratory conditions on small specimens. Reference [79] reports different values for the effective depth than what can be calculated from the technical drawings. The values from the drawings are used for the database. The ratio *a_v_*/*d* reported in [117] is 2.7. For the database entries, the size of the support plate measured from the technical drawings is used, and the effective depth is calculated assuming a cover of 10 mm. These assumptions result in *a_v_*/*d* = 2.83; the value of *a_v_*/*d* = 2.7 can’t be reverse-engineered based on the available information. Singh and Jain [4] mention that the smallest dimension of the cross-section should be at least three times the length of the longest fiber in the mix. As can be seen in the database, many experiments do not fulfil this requirement. Regardless of their comment, Singh and Jain proceeded to test specimens that do not fulfil this requirement, for ease of comparison to other test results.

The concrete compressive strength in the database is *f_c,cyl_*, the average concrete compressive strength as measured on cylinders. When the compressive strength is reported from cube specimens, the conversion *f_c,cyl_* = 0.85*f_c,cube_* is used. Reference [102] does not give the concrete compressive strength, but uses 3 ksi (21 MPa) in the presented calculation example. Therefore, the value of *f_c,cyl_* is reported as 21 MPa. Reference [119] does not report the concrete compressive strength. Normal strength concrete of *f_c,cyl_* = 30 MPa is assumed. References [50] and [112] used self-consolidating concrete. For references where the maximum aggregate size is not reported [52,74,82,91,109,115,119,120], a standard laboratory mix with *d_a_* = 10 mm is assumed. References [52,86,114,115,124,127] do not report the yield strength of the steel. For these cases *f_y_* = 420 MPa is assumed. For [108], the yield strength at 0.2% strain from the stress-strain diagram is used for the database.

When the tensile strength of the fibers was not given [50,52,71,89,97,98,100,107,108,110,111,115], the value of *f_tenf_* = 1100 MPa was assumed. For recent references, this assumption is reasonable, as this value is common for commercially available fibers. For the experiments by Batson [100] from 1972, it is only known that low-carbon steel was used for the fibers, but the tensile strength of the fibers is not known. The reported tensile strength for fibers by Ashour et al. [59] is smaller than for any other reference. The same value is reported in the paper in MPa and psi units, which seems to exclude a typing error in the reference. Reference [120] used recycled steel fibers. The properties of these fibers were not discussed in this reference, but for the database entries, reference [128] was consulted. Reference [123] does not report on the fiber type and properties. Therefore, standard commercially available hooked fibers were assumed. For the references where the amount of fibers is given as a mass, the fiber volume fraction is calculated by dividing the mass by 7800 kg/m^3^. When the concrete mix contained a combination of fibers [83], the reported fiber properties are weighted averages of the different fibers. Experiment B59 by [99] contained fibers only in the bottom 90 mm of the cross-section.

The results are given in terms of the sectional shear force at failure *V_utot_*, which includes the contribution of the self-weight, as well as in terms of the failure mode. Since this database includes the contribution of the self-weight, the shear at failure from this database may differ from what is reported in the original reference. For small specimens, the effect is small. For lightweight specimens [68,84], the density as reported in the original reference is taken into account to calculate the contribution of the self-weight. When this value was not reported in the original reference [94], a self-weight of 17 kN/m^3^ was assumed. In some references [81], the sectional shear force at failure *V_max_* or the applied load at failure *P_max_* is not included. Where possible [119,123], the load-displacement diagrams are used to read off this value. When this information was not presented, the experiments were not included in the database for lack of vital information. There is a factor 2 difference between the shear stress at failure *v_max_* in [102] and the value I calculated based on the size of the cross-section and the sectional shear at failure *V_max_*. The database contains this calculated value. What [118] reports as the shear force *V_max_* is actually *P_max_*, as one can see when calculating *v_max_*. The following abbreviations are used for the reported failure modes: B (bond failure of longitudinal reinforcement), DT (diagonal tension), NA (the failure mode for the individual experiment is not given in the original reference, but the text mentions that all experiments resulted in shear failure), S (shear failure), SC (shear-compression failure), S-FL (shear-flexure), ST (shear-tension), and Y (yielding of reinforcement).

#### 2.2.2. Parameter Ranges in Database

This section evaluates the distribution of the values of parameters over the database, in terms of range and shape of the distribution. Table 2 gives the ranges of key parameters in the database. These ranges show that the maximum height that has been tested (1220 mm) is relatively small to evaluate the size effect in shear [62,129,130,131,132,133]. The fiber types that occur in the database are: hooked, crimped, straight smooth, mixed (hooked + straight), fibers with a flat end, flat fibers, round fibers, mill-cut fibers, fibers of straight mild steel, brass-coated high strength steel fibers, chopped fibers with butt ends, recycled fibers, and corrugated fibers. The most frequently used fibers in the database are hooked (63% of all gathered experiments), crimped (22% of experiments), and straight smooth (3%).

**Figure 1:**
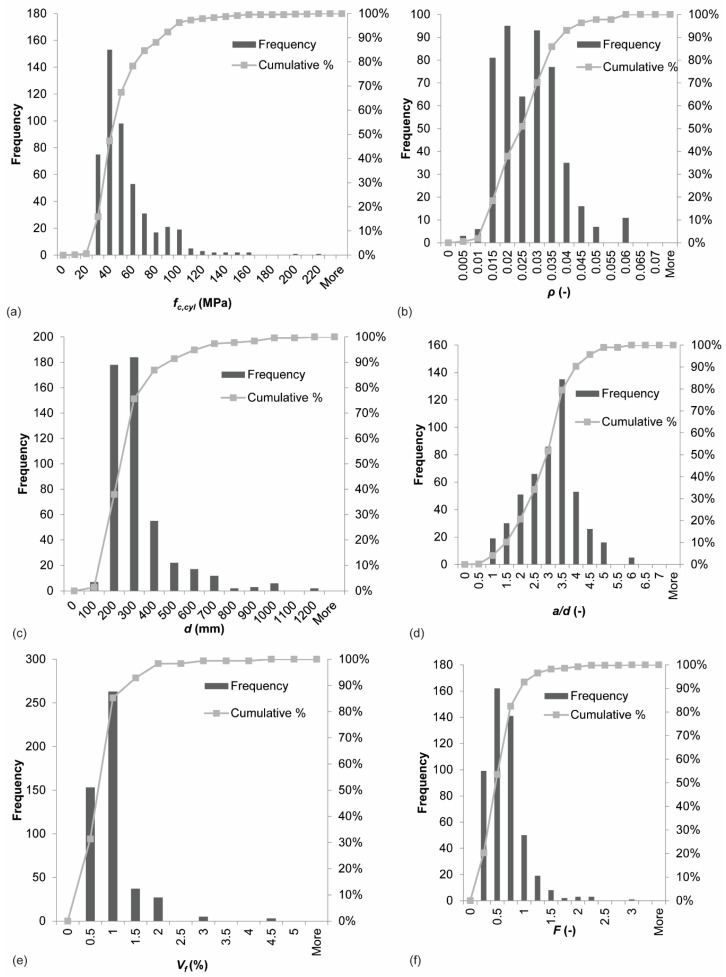
Distribution of parameters in database: (**a**) concrete compressive strength *f_c,cyl_*; (**b**) reinforcement ratio *ρ*; (**c**) effective depth *d*; (**d**) shear span to depth ratio *a*/*d*; (**e**) fiber volume fraction *V_f_*; and (**f**) fiber factor *F*.

Figure 1 shows the distribution of a selection of key parameters in the database. In terms of concrete compressive strength, Figure 1a shows that the results in the database are concentrated in the range of normal strength concrete, with some outliers for high and ultra-high strength concrete. For the reinforcement ratio, one can observe in Figure 1b that most specimens have large amounts of longitudinal steel, as typical for shear experiments where extra tension reinforcement is used to avoid a bending moment failure. The experiments are uniformly distributed in the range from 1.5–3.5% reinforcement. The database shows crowding in the range of small effective depths, see Figure 1c. The experiments are normally distributed in terms of shear span to depth ratio, see Figure 1d, with *a*/*d* = 3.5 as the most frequently used shear span. The histogram of the fiber volume fraction *V_f_*, Figure 1e, shows crowding in the range of 0.5–1.5%. This observation is not surprising, as these fractions are practical values: these fractions result in workable mixes, and serve the purpose of partially (not fully) replacing the mild steel reinforcement. Similarly, the observations for the histogram of the fiber factor *F* in Figure 1f reflect practical considerations and workability of SFRC.

## 3. Results

### 3.1. Parameter Studies

**Figure 2:**
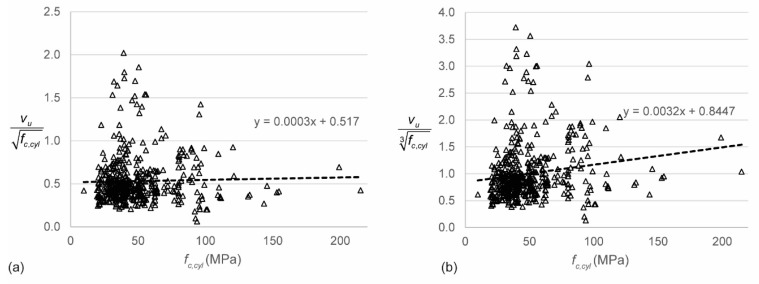
Normalized shear stresses to the concrete compressive strength: (**a**) normalized to the square root; (**b**) normalized to the cube root.

First, the raw data from the database are used to analyze the effect of different experimental parameters on the outcome (sectional shear stress at failure as a result of self-weight and applied load). To eliminate the influence of the concrete compressive strength *f_c,cyl_* on the parameter studies, normalized shear stresses are used. There is, however, quite some disagreement in the literature on the effect of the concrete compressive strength on the shear capacity [134]: should we normalize the shear stress with respect to the square or cube root of the concrete cylinder’s compressive strength? Therefore, I analyzed the normalized shear stress to both the square and cube root of the concrete as a function of the concrete compressive strength. Figure 2 shows the relation between the normalized shear stress and the concrete compressive strength *f_c,cyl_*. These results show that the shear stress should be normalized with respect to the square root of *f_c,cyl_*. The influence of different parameters will thus be studied as a function of the shear stress normalized to the square root of *f_c,cyl_*.

**Figure 3:**
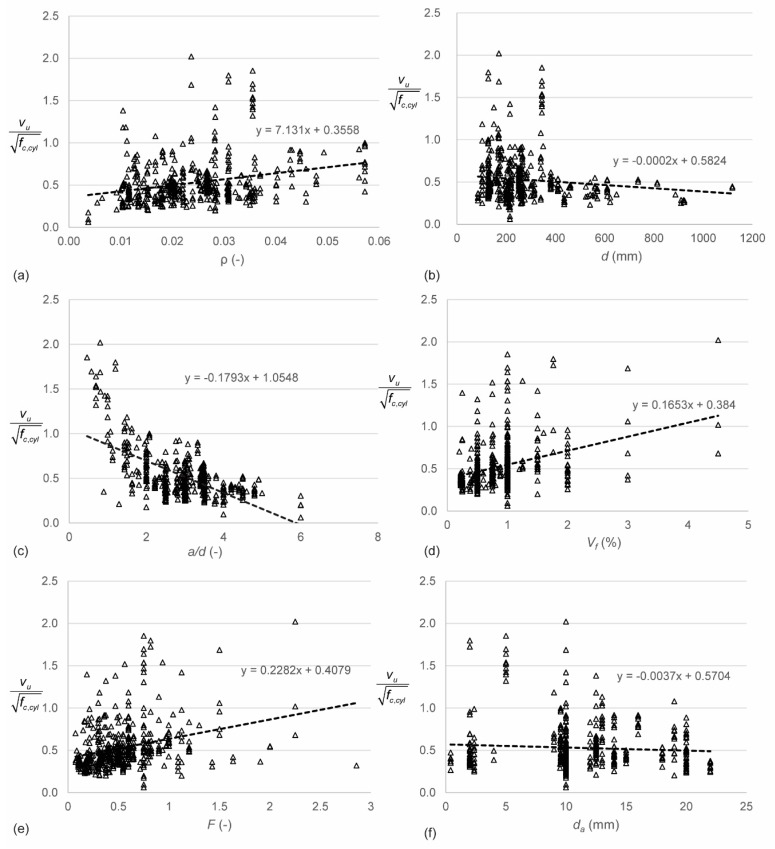
Parameter studies based on normalized shear stresses for all entries in database, influence of (**a**) longitudinal reinforcement ratio *ρ*; (**b**) effective depth *d*; (**c**) shear span to depth ratio *a*/*d*; (**d**) fiber volume fraction *V_f_*; (**e**) fiber factor *F*; and (**f**) maximum aggregate size *d_a_*.

Figure 3 gives an overview of the most important parameters and their influence on the shear stress normalized to the square root of *f_c,cyl_*. Figure 3a shows the influence of the reinforcement ratio *ρ*. Larger reinforcement ratios result in larger normalized shear capacities. This observation is expected, since larger reinforcement ratios result in a larger dowel action capacity [21,135,136], and thus a larger shear capacity. Figure 3b shows the influence of the effective depth *d* on the normalized shear stress. In reinforced concrete, the so-called size effect in shear [62,129,130,131,137,138] is known: the shear stress at failure reduces as the effective depth increases. The analysis of the database shows a small size effect. However, very few experiments on specimens with larger depths are available, as shown in Figure 1c. More experiments are necessary to study the size effect in SFRC. Figure 3c shows the influence of the shear span to depth ratio in terms of *a*/*d*. Note that the linear relation plotted on the graph is presented for consistency with the other figures, but does not accurately present the relation between *a*/*d* and the normalized shear strength. These results show that, just as for reinforced concrete beams, the shear capacity for specimens with *a*/*d* ≤ 2.5 increases for a decrease in *a*/*d*. The development of a compressive strut or arch between the point of application of the load and the support increases the shear capacity through the shear-carrying mechanism of arching action [139,140,141]. This influence can also be expressed as a function of the clear shear span to depth ratio *a_v_*/*d* and the generalized expression *M*/*Vd*. Since almost all experiments in the database are three- or four-point bending tests, the difference between *a*/*d* and *M*/*Vd* lies only in the contribution of the self-weight to *M* and *V*. For small specimens, this effect is negligible. For the current database therefore, the difference between the influence of *a*/*d* and *M*/*Vd* is negligible [142]. The parameter *a_v_*/*d* has a slightly larger influence on the normalized shear stress than *a*/*d*. This observation can be explained by the geometries used for deep beams in the database.

Figure 3d shows the relation between the normalized shear capacity and the fiber volume fraction *V_f_*. The normalized shear stress increases as the fiber volume fraction increases. The reason for this observation is the tension carried by the fibers across the crack. Figure 3e shows the relation between the fiber factor *F* and the normalized shear stress. Comparing Figure 3d,e shows that using the fiber factor *F* is an improvement as compared to using only the fiber volume fraction *V_f_*: less scatter is observed. Other properties of the fibers that were studied [142] were the aspect ratio *l_f_*/*d_f_* and the fiber tensile strength *f_tenf_*. The influence of the aspect ratio is similar to the influence of the fiber factor *F*, with the difference that the scatter on the plot with the fiber factor is smaller than for the plot with the aspect ratio. Small increases in the normalized shear strength were found for increases in the fiber tensile strength *f_tenf_*. Since the fibers typically do not reach their tensile strength, this observation is not surprising. Figure 3f shows the influence of the maximum aggregate size *d_a_* on the normalized shear strenght. The data show a minor decrease in normalized shear strength for increasing maximum aggregate size. Larger aggregates improve the aggregate interlock capacity [143,144], and it is often assumed that using smaller aggregates in small specimens is a conservative approach. For SFRC, however, smaller aggregates result in a more uniform concrete mix with a better bond between the fibers and the concrete.

### 3.2. Comparison to Code Predictions

The experimental shear capacities from the database are then compared to the shear capacities predicted by the code equations and equations proposed in the literature. A difficulty here lies in the definition of the tensile strength of the SFRC, which is based on different experiments depending on local or national practice. As such, it is not possible to build a database containing all values that quantify the tensile behavior of the SFRC, as none of the references report on the outcome of all possible tension tests. As a result, the equations proposed in the literature that were selected for this study depend as much as possible on the concrete compressive strength instead of on the tensile strength.

**Figure 4:**
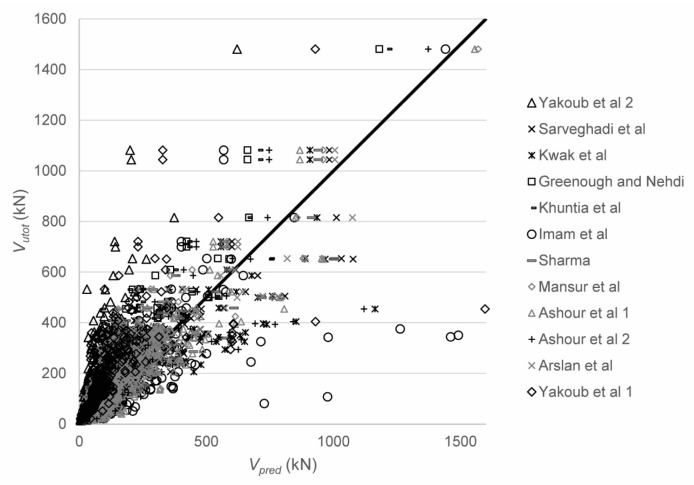
Comparison between experimental and predicted shear capacities for 12 methods from the literature.

In a first step, the shear capacity was predicted with 12 sets of equations in total: Sarveghadi et al. [28], Kwak et al. [48], Greenough and Nehdi [50], Khuntia et al. [51], Imam et al. [63], Sharma [52], Mansur et al. [54], Ashour et al. [59]—first equation, Ashour et al. [59]—second set of equations, Arslan et al. [60], Yakoub [64]—first set of equations, and Yakoub [64]—second set of equations. Table 1 contains all expressions. The expression by Greenough and Nehdi [50] uses the reinforcement ratio *ρ* as a percentage instead of as a number. Figure 4 shows the comparison between tested and predicted results, with the statistical properties of *V_utot_*/*V_pred_* in Table 3. Parametric studies for the influence of the different parameters are reported elsewhere [142]. Since not all proposed equations are (explicitly) valid for deep beams, the results for slender beams only are given in Table 4. For all datapoints, the expressions by Kwak et al. [48] result in the smallest coefficient of variation on the ratio of tested to predicted shear capacities and the mean value of tested to predicted shear capacity closest to 1.00, see Table 3. When only the slender beams are considered, the expressions by Arslan et al. [60] result in the smallest coefficient of variation on the tested to predicted shear capacities, combined with an average value of tested to predicted shear capacity close to 1.00 (1.04), see Table 4. In general, the scatter on the tested to predicted shear capacities is high. None of the expressions predicted in the literature is based on a mechanical model that studies the shear-carrying capacity of SFRC based on the mechanisms of shear transfer [18]. The expressions are (semi)-empirical, and thus depend on the database of experiments they were originally derived from. When developing a larger database, as part of this work, the equations do not perform well.

Next, the experimental shear capacities are compared to the code predictions. The code equations that were used for the predictions are the French recommendations [10], the German guideline [11], the *fib* 2010 Model Code [39], and the RILEM recommendations [66]. The predicted shear capacities with the Italian guide [12] are the same as with the *fib* 2010 Model Code [39]; *V_min_* never exceeds the shear capacity of the fiber reinforced concrete. Each of these codes requires the determination of the tensile strength according to experiments described in the respective codes. Since these results are not available in the reported experiments, except for the experiments carried out in the country where the code is valid, the properties had to be calculated. For determination of the tensile strength $f_{cfIk,L2}^{f}$ in the German guideline, the expression from Thomas [145] is used:

(57) $$f_{spfc}=0.63\sqrt{f_{cuf}}+0.288\times F\sqrt{f_{cuf}}+0.052\times F$$

To determine $f_{ctR,u}^{f}$, the value of $k_{F}^{f}=0.5$ for shear is used. The value of *C_Rd,c_* = 0.15 is used together with the German guideline, to reflect the German National Annex to the Eurocode, whereas *C_Rd,c_* = 0.18 is used together with the *fib* Model Code provisions and RILEM provisions. For determining *f_Ftuk_* as used in the *fib* Model Code, the value of $f_{ctR,u}^{f}$ from the German code is used. When comparing to the RILEM provisions, it is assumed that *f_Rk,_*_4_ = *f_spfc_* according to Equation (57). For all of the expressions based on the Eurocode shear provisions, the limitation of *ρ* ≤ 2% was removed, so that the heavily reinforced beams from the database could be evaluated as well.

**Figure 5:**
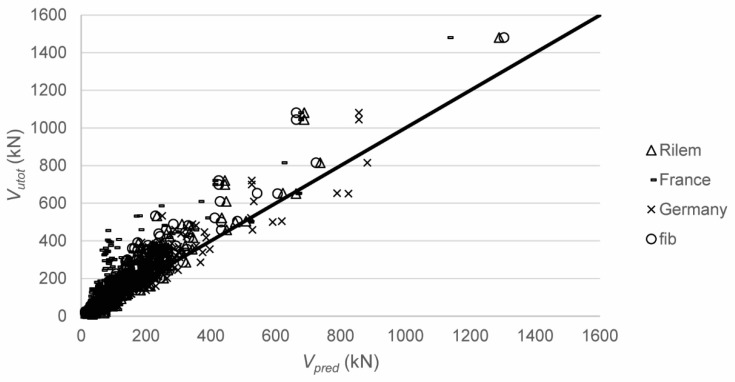
Comparison between tested and predicted shear capacities with the code formulas.

Figure 5 shows the comparison between the tested and predicted shear capacities according to the code equations. For the code equations that are based on the provisions from NEN-EN 1992-1-1:2005 [7], the reduction factor *β* = *a_v_*/*2d* for 0.5*d* ≤ *a_v_* ≤ *2d* is used on the externally applied load but not on the self-weight, to find the sectional shear force at the support *V_utot_*. Table 5 shows the statistical properties of the ratio of the tested to predicted shear capacities. This comparison shows a large scatter on the ratio of experimental to predicted values. For design purposes, the code prediction methods based on the Eurocode shear expression provide reasonable results (with coefficients of variation on the ratio of tested to predicted results of 27–29%). These proposed code equations tend to perform better than the equations proposed in the literature. Full parametric studies based on the tested to predicted shear capacities can be found elsewhere [142].

## 4. Discussion

None of the currently available methods properly describe the behavior of SFRC beams failing in shear, as none of the currently available methods describe the influence of adding steel fibers on the shear-carrying mechanisms: capacity in the compression zone, aggregate interlock, dowel action, residual tension, the contribution of the fibers across the crack, and arching action. This study shows the need for theoretical work that address the different shear-carrying mechanisms in SFRC and its crack kinematics. The large scatter on the ratios of tested to predicted shear capacities found in this study show that the currently available expressions do not describe the shear capacity of SFRC in a satisfactory manner. The code expressions based on the Eurocode are conservative, have smaller scatter as compared to the other expressions, and it seems that these can be used currently for the purpose, as practitioners wait for improved expressions.

An analysis of the ranges of parameters used in the experiments from the literature shows that the majority of tested specimens are small, heavily reinforced for flexure, and tested in three- or four-point bending. Such beams are typical for shear experiments. One may however question how representative such specimens are for actual structural elements. In my opinion, laboratory specimens provide valuable insight into the behavior of SFRC beams failing in shear, but cannot address all open questions. For the implementation of SFRC beams and one-way slabs in buildings and bridges, full-size beams and girders should be designed, and their performance should be evaluated experimentally. Full-size specimens are also required to study the size effect in shear for SFRC.

In earlier work [146], I followed the approach of adding a separate term to quantify the contribution of the steel fibers, in addition to the capacity of the concrete expressed by using the Critical Shear Displacement Theory [147]. This approach is followed by a number of the currently available codes and equations proposed in the literature. However, a further study of the influence of adding steel fibers to the concrete on the shear capacity and the individual shear-carrying mechanisms [18] led me to the conclusion that isolating the contribution of the fibers in a separate, single term is theoretically not correct. The influence of the fibers on all shear-carrying mechanisms should be quantified theoretically, and then evaluated experimentally (for example, with digital image correlation analysis [148,149,150]).

A better understanding of how steel fibers improve the shear resistance of SFRC is important to allow a wider use of SFRC in structural applications. Likewise, a better understanding of the contribution of steel fibers to the shear capacity can result in optimization of cross-sections, a more optimal and economical use of materials, and thus more sustainable designs.

## 5. Summary and Conclusions

One of the barriers for more widespread use of steel fiber reinforced concrete (SFRC) in structural applications, such as beams and girders where part of the stirrups are replaced by fibers, or slabs without stirrups, is the lack of understanding of the shear-carrying behavior. This lack of understanding is reflected by the fact that only a handful of national codes or guidelines contain expressions to quantify the shear capacity of SFRC. This study evaluates the currently available code provisions and equations proposed in the literature for the shear capacity of SFRC elements without stirrups against a database of 488 experimental results from the literature. This study provides an inventory of the current knowledge, identifies the gaps, and proposes a way forward for research on the shear capacity of SFRC elements.

Analyzing the available experimental results from the database resulted in the following conclusions:

- Most experiments are carried out on small specimens.
- There is a lack of experiments on SFRC beams with a large depth, which is necessary to evaluate the size effect in shear.
- Most specimens have a large reinforcement ratio, which is common for shear tests to avoid a flexural failure but does not correspond to actual designs.
- Experiments on deep and slender beams are available to evaluate the influence of the shear span to depth ratio.
- The majority of the specimens are cast with normal strength concrete.
- Most of the fiber volume fractions in the specimens lie between 0.5–1.5% as this range contains practical and workable amounts of fibers and fulfils the aim of partially replacing the mild steel shear reinforcement. The full range of fiber volume fractions in the database is 0.2–4.5%.
- Historically, different fiber types have been included in experiments. Nowadays, the most commonly used and commercially available fibers are hooked-end fibers. This practice is reflected in the database: 63% of the reported experiments use hooked-end fibers.

Then, parameter studies were carried out based on the available experimental results from the database, which led to the following observations:

- An analysis of the data shows that the shear stresses should be normalized to the square root of the concrete compressive strength, as this ratio shows a smaller relation to the concrete compressive strength than the cube root of the concrete compressive strength.
- The normalized shear strength increases as the reinforcement ratio increases, which can be explained by the larger dowel action for larger amounts of reinforcement.
- The data show a small decrease for the normalized shear strength as the effective depth increases. Not enough experimental results on large SFRC beams are available to study the size effect in shear in SFRC.
- The influence of the shear span to depth ratio on the normalized shear strength is similar in SFRC as in reinforced concrete. The higher shear strength for small values of the shear span to depth ratio is the result of arching action.
- The normalized shear strength increases as the fiber volume fraction increases. The normalized shear strength increases as the fiber factor increases. These observations are expected, since the contribution of the fibers improves the shear capacity. There is less scatter on the influence of the fiber factor than on the influence of the fiber volume fraction, which justifies the use of the fiber factor in expressions and code equations.
- The normalized shear strength decreases as the maximum aggregate size increases. This observation in contrary to what happens in reinforced concrete, where larger aggregates improve the aggregate interlock capacity and thus the shear capacity. In SFRC, smaller aggregates result in a more uniform mix, and a better bond between the concrete matrix and the steel fibers, which enhances the shear capacity.

For the comparison between the experimental shear capacities and the capacities predicted by the currently available codes and equations proposed in the literature, the following conclusions result:

- National codes and guidelines are based on specific methods for determining the tensile strength of the SFRC, and these methods differ internationally. As such, none of the experiments available in the literature report on all values of the tensile strength that are required for determining the tensile strength in the various expressions.
- The ratio of tested to predicted shear capacities shows large scatter. When all experiments are considered, the expression by Kwak et al. results in the best performance. When only slender beams are considered, the expression by Arslan et al. results in the best performance.
- The code equations based on the Eurocode shear expressions have a coefficient of variation between 27% and 29% and a slightly conservative value of the average ratio of the tested to predicted shear capacity. As such, these equations can be used until better proposals are available.

The analysis in this work shows the need for a better understanding of the shear capacity of SFRC. An analysis of the influence of the steel fibers on all shear-carrying mechanisms seems necessary. A better understanding of the shear-carrying mechanisms is necessary to allow a more widespread use of SFRC in structural elements, and an optimization of designs.

## Figures and Tables

**Table 1:** Shear prediction equations from literature and available codes.

Authors	Reference	Expression	
Sarveghadi et al.	[28]	$V_{u}=\left[\rho +\frac{\rho }{v_{b}}+\frac{1}{\frac{a}{d}}\left(\frac{\rho f_{t}{}^{\prime }\left(\rho +2\right)\left(f_{t}{}^{\prime }\frac{a}{d}-\frac{3}{v_{b}}\right)}{\frac{a}{d}}+f_{t}{}^{\prime }\right)+v_{b}\right]b_{w}d$	(2)
		$f_{t}{}^{\prime }=0.79\sqrt{f_{c}{}^{\prime }}$	(3)
		$v_{b}=0.41\tau F$ with *τ* = 4.15 MPa	(4)
Kwak et al.	[48]	$V_{u}=\left[3.7ef_{spfc}^{2/3}{\left(\rho \frac{d}{a}\right)}^{1/3}+0.8v_{b}\right]b_{w}d$	(5)
		$f_{spfc}=\frac{f_{cuf}}{\left(20-\sqrt{F}\right)}+0.7+1.0\sqrt{F}$ in MPa	(6)
		$e=\left\{ \begin{array}{l} 1\;\mathrm{for}\;\frac{a}{d}>3.4\\ 3.4\frac{d}{a}\;\mathrm{for}\;\frac{a}{d}\leq 3.4 \end{array}\right.$	(7)
Greenough and Nehdi	[50]	$V_{u}=\left[0.35\left(1+\sqrt{\frac{400}{d}}\right){\left(f_{c}{}^{\prime }\right)}^{0.18}{\left(\left(1+F\right)\rho \frac{d}{a}\right)}^{0.4}+0.9\eta_{o}\tau F\right]b_{w}d$	(8)
Kuntia et al.	[51]	$V_{u}=\left[\left(0.167+0.25F\right)\sqrt{f_{c}{}^{\prime }}\right]b_{w}d$	(9)
Sharma	[52]	$V_{u}=\left(\frac{2}{3}\times 0.8\sqrt{f_{c}{}^{\prime }}{\left(\frac{d}{a}\right)}^{0.25}\right)b_{w}d$	(10)
Mansur et al.	[54]	$V_{u}=V_{c}+\sigma_{tu}b_{w}d$	(11)
		$V_{c}=\left(0.16\sqrt{f_{c}{}^{\prime }}+17.2\frac{\rho Vd}{M}\right)b_{w}d\leq 0.29\sqrt{f_{c}{}^{\prime }}b_{w}d$	(12)
		$\sigma_{tu}=3.2\eta_{o}\eta_{l}F\tau \;\mathrm{with}\;\tau =2.58\;\mathrm{MPa}$	(13)
		$\eta_{l}=1-\frac{\tanh\left(\beta \frac{l_{f}}{2}\right)}{\beta \frac{l_{f}}{2}}$	(14)
		$\beta =\sqrt{\frac{2\pi G_{m}}{E_{f}A_{f}\ln\left(\frac{S}{r_{f}}\right)}}$	(15)
		$S=25\sqrt{\frac{d_{f}}{V_{f}l_{f}}}$	(16)
Ashour et al.	[59]	$V_{u}=\left[\left(0.7\sqrt{f_{c}{}^{\prime }}+7F\right)\frac{d}{a}+17.2\rho \frac{d}{a}\right]b_{w}d$	(17)
		$V_{u}=\left[\left(2.11\sqrt[{3}]{f_{c}{}^{\prime }}+7F\right){\left(\rho \frac{d}{a}\right)}^{0.333}\right]b_{w}d\;\mathrm{for}\;\frac{a}{d}\geq 2.5$	(18)
		$V_{u}=\left[\left(\left(2.11\sqrt[{3}]{f_{c}{}^{\prime }}+7F\right){\left(\rho \frac{d}{a}\right)}^{0.333}\right)\frac{2.5}{\frac{a}{d}}+v_{b}\left(2.5-\frac{a}{d}\right)\right]b_{w}d\;\mathrm{for}\;\frac{a}{d}<2.5$	(19)
Arslan et al.	[60]	$V_{u}=\left[\left(0.2{\left(f_{c}{}^{\prime }\right)}^{2/3}\frac{c}{d}+\sqrt{\rho \left(1+4F\right)f_{c}{}^{\prime }}\right)\sqrt[{3}]{\frac{3}{\frac{a}{d}}}\right]b_{w}d$	(20)
		${\left(\frac{c}{d}\right)}^{2}+\left(\frac{600\rho }{f_{c}{}^{\prime }}\right)\left(\frac{c}{d}\right)-\frac{600\rho }{f_{c}{}^{\prime }}=0$	(21)
Imam et al.	[63]	$V_{u}=\left[0.6\psi \sqrt[{3}]{\omega }\left({\left(f_{c}{}^{\prime }\right)}^{0.44}+275\sqrt{\frac{\omega }{{\left(\frac{a}{d}\right)}^{5}}}\right)\right]b_{w}d$	(22)
		$\psi =\frac{1+\sqrt{\frac{5.08}{d_{a}}}}{\sqrt{1+\frac{d}{25d_{a}}}}$	(23)
		ω=ρ(1+4F)	(24)
Yakoub	[64]	$V_{u}=\left[0.83\xi \sqrt[{3}]{\rho }\left(\sqrt{f_{c}{}^{\prime }}+249.28\sqrt{\frac{\rho }{{\left(\frac{a}{d}\right)}^{5}}}+0.405\frac{l_{f}}{d_{f}}V_{f}R_{g}\frac{d}{a}\sqrt{f_{c}{}^{\prime }}\right)\right]b_{w}d\;\mathrm{for}\;\frac{a}{d}\leq 2.5$	(25)
		$V_{u}=\left[0.83\xi \sqrt[{3}]{\rho }\left(\sqrt{f_{c}{}^{\prime }}+249.28\sqrt{\frac{\rho }{{\left(\frac{a}{d}\right)}^{5}}}+0.162\frac{l_{f}}{d_{f}}V_{f}R_{g}\sqrt{f_{c}{}^{\prime }}\right)\right]b_{w}d\;\mathrm{for}\;\frac{a}{d}\geq 2.5$	(26)
		$\xi =\frac{1}{\sqrt{1+\frac{d}{25d_{a}}}}$	(27)
		$V_{u}=2.5\left(\frac{0.40}{1+1500\varepsilon_{x}}\times \frac{1300}{1000+s_{xe}}\right)\sqrt{f_{c}{}^{\prime }}\left(1+0.7\frac{l_{f}}{d_{f}}V_{f}R_{g}\right)\frac{d}{a}b_{w}d_{v}\;\mathrm{for}\;\frac{a}{d}\leq 2.5$	(28)
		$V_{u}=\left(\frac{0.40}{1+1500\varepsilon_{x}}\times \frac{1300}{1000+s_{xe}}\right)\sqrt{f_{c}{}^{\prime }}\left(1+0.7\frac{l_{f}}{d_{f}}V_{f}R_{g}\right)b_{w}d_{v}\;\mathrm{for}\;\frac{a}{d}\geq 2.5$	(29)
		$d_{v}=\max\left(0.9d,0.72h\right)$	(30)
		$\varepsilon_{x}=\frac{\frac{M}{d_{v}}+V}{2E_{s}A_{s}}$	(31)
		$s_{xe}=\frac{35s_{x}}{16+d_{a}}\geq 0.85s_{x}\;\mathrm{and}\;s_{x}\approx d_{v}$	(32)
Association Française de Génie Civil	[10]	$V_{Rd}=V_{Rd,c}+V_{Rd,f}$	(33)
		$V_{Rd,c}=\frac{0.21}{\gamma_{cf}\gamma_{E}}f_{ck}^{1/2}b_{w}d$	(34)
		$V_{Rd,f}=\frac{A_{vf}\sigma_{Rd,f}}{\tan\theta }$	(35)
		$\sigma_{Rd,f}=\left\{ \begin{array}{l} \frac{1}{K\gamma_{cf}}\frac{1}{w_{\lim}}\int_{0}^{w_{\lim}}\sigma_{f}\left(w\right)dw\;\mathrm{for}\;\mathrm{strain}\;\mathrm{softening}\;\mathrm{or}\;\mathrm{low}\;\mathrm{strain}\;\mathrm{hardening}\\ \frac{1}{K\gamma_{cf}}\frac{1}{\varepsilon_{\lim}-\varepsilon_{el}}\int_{\varepsilon_{el}}^{\varepsilon_{\lim}}\sigma_{f}\left(\varepsilon \right)d\varepsilon \;\mathrm{for}\;\mathrm{high}\;\mathrm{strain}\;\mathrm{hardening} \end{array}\right.$	(36)
		$w_{\lim}=\max\left(w_{u},w_{\max}\right)$	(37)
		$\varepsilon_{\lim}=\max\left(\varepsilon_{u},\varepsilon_{\max}\right)$	(38)
		$A_{vf}=b_{w}z$	(39)
DAfStB	[11]	$V_{Rd,c}^{f}=V_{Rd,c}+V_{Rd,cf}$	(40)
		$V_{Rd,c}=\frac{C_{Rd,c}}{\gamma_{c}}k{\left(100\rho f_{ck}\right)}^{1/3}b_{w}d>V_{Rd,c,\min}$	(41)
		$V_{Rd,cf}=\frac{\alpha_{c}^{f}f_{ctR,u}^{f}b_{w}h}{\gamma_{ct}^{f}}$	(42)
		$f_{ctR,u}^{f}=k_{F}^{f}k_{G}^{f}0.37f_{cfIk,L2}^{f}$	(43)
		$k_{G}^{f}=1.0+0.5A_{ct}^{f}\leq 1.7$	(44)
		$A_{ct}^{f}=b_{w}\times \min\left(d,1.5m\right)$	(45)
		$k=1+\sqrt{\frac{200mm}{d}}$	(46)
RILEM	[66]	$V_{Rd}=V_{cd}+V_{fd}$	(47)
		$V_{cd}=0.12k{\left(100\rho f_{ck}\right)}^{\frac{1}{3}}b_{w}d$	(48)
		$V_{fd}=0.7k_{f}k\tau_{fd}b_{w}d$	(49)
		$k_{f}=1+n\left(\frac{h_{f}}{b_{w}}\right)\left(\frac{h_{f}}{d}\right)\leq 1.5$	(50)
		$n=\frac{b_{f}-b_{w}}{h_{f}}\leq 3\;\mathrm{and}\;n\leq \frac{3b_{w}}{h_{f}}$	(51)
		$\tau_{fd}=0.12f_{Rk,4}$	(52)
*fib*	[39]	$V_{Rd}=V_{Rd,f}=\frac{C_{Rd,c}}{\gamma_{c}}k{\left(100\rho \left(1+7.5\frac{f_{Ftuk}}{f_{ctk}}\right)f_{ck}\right)}^{1/3}b_{w}d$	(53)
		$f_{ctk}=\left\{ \begin{array}{l} 0.3{\left(f_{ck}\right)}^{2/3}\;\mathrm{for}\;\mathrm{concrete}\;\mathrm{grades}\;\leq \;C50\\ 2.12\ln\left(1+0.1\left(f_{ck}+8MPa\right)\right)\mathrm{for}\;\mathrm{concrete}\;\mathrm{grades}\;>\;C50 \end{array}\right.$	(54)
CNR-DT	[12]	$V_{Rd}=V_{Rd,f}\geq V_{min}$	(55)
		$V_{min}=0.035k^{3/2}f_{ck}^{1/2}b_{w}d$	(56)

**Table 2:** Ranges of parameters in database.

Parameter	Min	Max
*b*_w_ (mm)	50	610
*h* (mm)	100	1220
*d* (mm)	85	1118
*l_span_* (mm)	204	7823
*a* (mm)	102	3912
*a_v_* (mm)	52	3747
*ρ* (%)	0.37%	5.72%
*f_y_* (MPa)	276	900
*a*/*d* (-)	0.46	6
*a_v_*/*d* (-)	0.20	5.95
*d_a_* (mm)	0.4	22
*f_c,cyl_* (MPa)	9.8	215
*V_f_* (%)	0.2	4.5
*l_f_*/*d_f_* (-)	25	191
*f_tenf_* (MPa)	260	4913
*F* (-)	0.075	2.858

**Table 3:** Statistical properties of *V_utot_*/*V_pred_* for all 488 datapoints, with AVG = average of *V_utot_/V_pred_*, STD = standard deviation on *V_utot_/V_pred_*, and COV = coefficient of variation of *V_utot_/V_pred_*.

Model	AVG	STD	COV	Min	Max
Sarveghadi et al. [28]	1.03	0.29	28%	0.23	2.49
Kwak et al. [48]	1.01	0.28	27%	0.27	2.39
Greenough and Nehdi [50]	1.34	0.48	36%	0.31	3.11
Khuntia et al. [51]	1.81	0.85	47%	0.18	6.53
Imam et al. [63]	0.97	0.36	37%	0.06	2.51
Sharma [52]	1.24	0.49	39%	0.18	3.59
Mansur et al. [54]	1.30	0.60	46%	0.15	3.85
Ashour et al. [59] 1	1.08	0.38	35%	0.24	3.14
Ashour et al. [59] 2	1.29	0.37	29%	0.31	3.22
Arslan et al. [60]	1.17	0.37	31%	0.43	3.24
Yakoub [64] 1	1.90	0.76	40%	0.28	7.50
Yakoub [64] 2	2.97	1.37	46%	0.51	17.48

**Table 4:** Statistical properties of *V_test_*/*V_pred_* for 352 datapoints with *a*/*d* ≥ 2.5, with AVG = average of *V_utot_/V_pred_*, STD = standard deviation on *V_utot_/V_pred_*, and COV = coefficient of variation of *V_utot_/V_pred_*.

Model	AVG	STD	COV	Min	Max
Sarveghadi et al. [28]	1.02	0.29	28%	0.23	2.20
Kwak et al. [48]	1.06	0.28	26%	0.27	2.39
Greenough and Nehdi [50]	1.20	0.37	30%	0.31	3.11
Khuntia et al. [51]	1.53	0.48	31%	0.18	4.03
Imam et al. [63]	1.07	0.31	29%	0.32	2.51
Sharma [52]	1.11	0.33	30%	0.18	2.28
Mansur et al. [54]	1.12	0.42	38%	0.15	3.57
Ashour et al. [59] 1	1.15	0.40	35%	0.24	3.14
Ashour et al. [59] 2	1.35	0.35	26%	0.47	3.22
Arslan et al. [60]	1.04	0.24	23%	0.43	1.97
Yakoub [64] 1	2.03	0.80	39%	0.62	7.50
Yakoub [64] 2	2.83	1.37	49%	0.61	17.48

**Table 5:** Statistical properties of *V_utot_*/*V_pred_* for all 488 datapoints, with AVG = average of *V_utot_/V_pred_*, STD = standard deviation on *V_utot_/V_pred_*, and COV = coefficient of variation of *V_utot_/V_pred_*.

Model	AVG	STD	COV	Min	Max
French code [10]	1.85	0.88	48%	0.22	5.95
German code [11]	1.12	0.31	27%	0.21	2.13
*fib* [39]	1.24	0.36	29%	0.30	2.33
RILEM [66]	1.16	0.33	29%	0.23	2.28

## List of Notations

*a*	shear span, distance between left of loading plate and left of support
*a_v_*	clear shear span, distance between face of loading plate and face of support
*b_w_*	web width
*c*	height of compression zone
*d*	effective depth
*d_a_*	maximum aggregate size
*d_f_*	fiber diameter
*d_v_*	shear depth
*e*	factor to take effect of shear span to depth ratio into account
*f_c_’*	specified concrete compressive strength
*f_c,cube_*	average measured concrete cube compressive strength
*f_c,cyl_*	average measured concrete cylinder compressive strength
$f_{cfIk,L2}^{f}$	characteristic value of post-cracking flexural strength for a deflection of 3.5 mm
*f_ck_*	characteristic concrete cylinder compressive strength
*f_ctk_*	characteristic tensile strength of concrete
$f_{ctR,u}^{f}$	uniaxial tensile strength of SFRC
*f_cuf_*	cube compressive strength of fiber reinforced concrete
*f_Ftuk_*	characteristic value of post-cracking strength for ultimate crack opening
*f_Rk,4_*	characteristic residual flexural strength for the ultimate limit state at a CMOD of 3.5 mm
*f_spfc_*	splitting tensile strength of fiber reinforced concrete
*f_t_’*	specified tensile strength of concrete mix
*f_tenf_*	tensile strength of the fibers
*f_y_*	yield strength of the reinforcement steel
*h*	height of cross-section
*h_f_*	height of flange
*k*	size effect factor
*k_f_*	factor that considers the contribution of flanges in T-sections (= 1 for rectangular sections)
$k_{F}^{f}$	factor that considers the orientation of the fibers
$k_{G}^{f}$	size factor, which accounts for the fact that fibers are better distributed in larger elements
*l_f_*	fiber length
*l_span_*	span length
*l_tot_*	total specimen length
*n*	parameter for effect of geometry of flanged sections
*r_f_*	fiber radius
*s_x_*	crack spacing
*s_xe_*	equivalent crack spacing factor
*v_max_*	shear stress at maximum sectional shear *V_max_*
*w_lim_*	limiting crack width
*w_max_*	maximum crack width permitted by the code
*w_u_*	ultimate crack width, i.e., the value attained at the ULS for resistance to combined stresses on the outer fiber under the moment exerted in this section
*v_b_*	shear strength attributed to fibers
*z*	internal lever arm
$A_{ct}^{f}$	effective area *b_w_* × *d*, with *d* limited to 1.5 m
*A_f_*	cross-sectional area of the fiber
*A_s_*	area of longitudinal tension reinforcement
*A_vf_*	shear area over which fibers contribute
B	failure of bond between concrete and longitudinal reinforcement
*C_Rd,c_*	calibration factor for the design shear capacity
DT	diagonal tension failure
*E_f_*	modulus of elasticity of the fibers
*E_s_*	modulus of elasticity of reinforcement steel
*F*	fiber factor
*G_m_*	matrix shear modulus
*K*	orientation coefficient
*M*	sectional moment
NA	the failure mode of the individual experiment is not given, but the text mentions that all experiments resulted in a shear failure
*P_max_*	maximum load in experiment
*R_g_*	geometry factor from Yakoub [64]: 0.83 for crimped fibers, 1.00 for hooked fibers, and 0.91 for round fibers
*S*	fiber spacing
S	shear failure
SC	shear-compression failure
S-FL	shear-flexure failure
ST	shear-tension failure
*V*	sectional shear force
*V_c_*	concrete contribution to shear capacity
*V_cd_*	design value of concrete contribution to shear capacity
*V_f_*	fiber volume fraction
*V_fd_*	design value of fiber contribution to shear capacity
*V_max_*	maximum sectional shear in experiment caused by applied load only (without self-weight)
*V_min_*	lower bound to the shear capacity
*V_pred_*	predicted shear capacity
*V_Rd_*	design shear capacity
*V_Rd,c_*	design shear capacity of the concrete contribution
$V_{Rd,c}^{f}$	design shear capacity of fiber reinforced concrete
*V_Rd,cf_*	design shear capacity of the fiber contribution, notation used in German guideline
*V_Rd,c,min_*	lower bound to the design shear capacity of the concrete contribution
*V_Rd,f_*	design shear capacity of the steel fiber contribution
*V_u_*	ultimate shear capacity
*V_utot_*	experimental shear capacity, including contribution from self-weight
Y	failure mode includes yielding of longitudinal reinforcement
$\alpha_{c}^{f}$	factor that accounts for the long term effects
*β*	fiber and matrix property factor developed by Cox [56]
*γ_c_*	concrete material factor
*γ_cf_*	concrete material factor, notation used in French guideline
$\gamma_{ct}^{f}$	partial factor for tensile strength of fiber reinforced concrete
*γ_E_*	additional safety factor
*ε_el_*	elastic strain
*ε_lim_*	limiting strain
*ε_max_*	maximum strain
*ε_u_*	ultimate strain at the ULS for bending combined with axial forces on the outer fiber under the moment exerted in the section
*ε_x_*	strain at mid-depth of the cross-section
*η_o_*	fiber orientation factor = 0.41 for fibers with a 3D random orientation, as derived by Romualdi and Mandel [151], but can be larger for members with thin webs
*η_l_*	a length factor used to account for the variability in the fiber embedment length across the cracking plane
*θ*	angle of compression strut
ξ	size effect factor from Bažant and Kim [62]
*ρ*	reinforcement ratio
*ρ_f_*	fiber bond factor: 0.5 for straight fibers, 0.75 for crimped fibers, 1 for hooked fibers
*σ_Rd,f_*	residual tensile strength of fiber reinforced cross-section
*σ_f_(ε)*	experimentally determined relation between stress in fiber concrete and strain
*σ_f_(w)*	experimentally determined relation between post-cracking stress and crack width *w*
*σ_tu_*	average stress at the ultimate limit state in the equivalent tensile stress block used for bending moment analysis of SFRC
*τ*	bond strength between fibers and matrix
*τ_fd_*	design value of bond strength between fibers and matrix
*ψ*	size effect factor from Imam et al. [63]
*ω*	reinforcement ratio that includes the effect of fibers

## Appendix A

**Table A1:** Database of experimental results from literature of SFRC beams with longitudinal reinforcement without stirrups failing in shear.

		Geometry							Concrete Mix		Fibers					Results
Reference	ID	*b_w_*	*h*	*d*	*l_span_*	*ρ*	*a/d*	*a_v_/d*	*d_a_*	*f_c,cyl_*	Fiber Type	*V_f_*	*l_f_/d_f_*	*f_tenf_*	*V_utot_*	Failure Mode
		(mm)	(mm)	(mm)	(mm)	(-)	(-)	(-)	(mm)	(MPa)		(%)	(-)	(MPa)	(kN)	
Singh & Jain 2014 [4]	D-I	150	300	251	1470	0.0267	3.49	3.09	12.5	28.1	hooked	0.75	65	1100	114	DT + ST + SC
	D-II	150	300	251	1470	0.0267	3.49	3.09	12.5	25.3	hooked	0.75	65	1100	80	DT + ST + SC
	E-I	150	300	251	1470	0.0267	3.49	3.09	12.5	27.9	hooked	1	65	1100	110	DT + ST + SC
	E-II	150	300	251	1470	0.0267	3.49	3.09	12.5	26.2	hooked	1	65	1100	124	DT + ST + SC
	F-I	150	300	251	1470	0.0267	3.49	3.09	12.5	28.1	hooked	1.5	65	1100	112	DT + ST + SC
	F-II	150	300	251	1470	0.0267	3.49	3.09	12.5	27.3	hooked	1.5	65	1100	132	DT + ST + SC
	G-I	150	300	251	1470	0.0267	3.49	3.09	12.5	27.5	hooked	0.5	80	1050	66	DT + ST + SC
	G-II	150	300	251	1470	0.0267	3.49	3.09	12.5	24.9	hooked	0.5	80	1050	78	DT + ST + SC
	H-I	150	300	251	1470	0.0267	3.49	3.09	12.5	27.8	hooked	0.75	80	1050	92	DT + ST + SC
	H-II	150	300	251	1470	0.0267	3.49	3.09	12.5	27.3	hooked	0.75	80	1050	102	DT + ST + SC
	I-I	150	300	251	1470	0.0267	3.49	3.09	12.5	26.3	hooked	1	80	1050	117	DT + ST + SC
	I-II	150	300	251	1470	0.0267	3.49	3.09	12.5	27.1	hooked	1	80	1050	105	DT + ST + SC
	K-I	150	300	251	1470	0.0267	3.49	3.09	12.5	53.4	hooked	0.75	65	1100	114	DT + ST
	K-II	150	300	251	1470	0.0267	3.49	3.09	12.5	54.1	hooked	0.75	65	1100	127	DT + ST
	L-I	150	300	251	1470	0.0267	3.49	3.09	12.5	53.2	hooked	1	65	1100	145	DT + ST
	L-II	150	300	251	1470	0.0267	3.49	3.09	12.5	55.3	hooked	1	65	1100	166	DT + ST + SC
	P-I	150	300	251	1470	0.0267	3.49	3.09	12.5	64.6	hooked	1.5	65	1100	196	DT + ST
	P-II	150	300	251	1470	0.0267	3.49	3.09	12.5	59.9	hooked	1.5	65	1100	161	DT + ST + SC
	AA-I	150	300	251	1470	0.0267	3.49	3.09	12.5	47.8	hooked	0.5	80	1050	128	DT + ST + SC
	AA-II	150	300	251	1470	0.0267	3.49	3.09	12.5	49.5	hooked	0.5	80	1050	153	DT + ST + SC
	M-I	150	300	251	1470	0.0267	3.49	3.09	12.5	55.3	hooked	0.75	80	1050	147	DT + ST + SC
	M-II	150	300	251	1470	0.0267	3.49	3.09	12.5	56.4	hooked	0.75	80	1050	179	DT + ST
	N-I	150	300	251	1470	0.0267	3.49	3.09	12.5	53.4	hooked	1	80	1050	129	DT + ST + SC
	N-II	150	300	251	1470	0.0267	3.49	3.09	12.5	51	hooked	1	80	1050	158	DT + ST
	R-I	150	300	251	1470	0.0267	3.49	3.09	12.5	27.8	crimped	1	50	1025	80	DT + ST + SC
	R-II	150	300	251	1470	0.0267	3.49	3.09	12.5	27.2	crimped	1	50	1025	79	DT + ST + SC
	U-I	150	300	251	1470	0.0267	3.49	3.09	12.5	27.6	crimped	1	85	1050	99	DT + ST + SC
	U-II	150	300	251	1470	0.0267	3.49	3.09	12.5	27.9	crimped	1	85	1050	82	DT + ST + SC
	W-I	150	300	251	1470	0.0267	3.49	3.09	12.5	34.7	crimped	1	50	1025	100	DT + ST + SC
	W-II	150	300	251	1470	0.0267	3.49	3.09	12.5	36.2	crimped	1	50	1025	101	DT + ST
	Z-I	150	300	251	1470	0.0267	3.49	3.09	12.5	37	crimped	1	85	1050	111	DT + ST
	Z-II	150	300	251	1470	0.0267	3.49	3.09	12.5	38.3	crimped	1	85	1050	105	DT + ST
Sahoo & Sharma	M-25-0.50	150	300	261	1800	0.0116	2.30	1.92	20.0	28.7	hooked	0.5	80	1100	144	S-FL
2014	M20-S-0.75	150	300	261	1800	0.0195	3.45	3.07	20.0	32.9	hooked	0.75	80	1100	109	S
[67]	M20-S-1	150	300	261	1800	0.0195	3.45	3.07	20.0	23.8	hooked	1	80	1100	94	S
	M20-S-1.25	150	300	261	1800	0.0195	3.45	3.07	20.0	24.1	hooked	1.25	80	1100	115	S
Shoaib, Lubell and Bindiganavile	L31	310	308	258	1548	0.0184	3.00	2.42	10.0	22	hooked	1	55	1100	204	S-FL
2015	L32	310	308	258	1548	0.0245	3.00	2.42	10.0	31	hooked	1	55	1100	299	S-FL
[68]	L62	300	600	550	3300	0.0119	3.00	2.73	10.0	30	hooked	1	55	1100	312	S-FL
Manju et al.	SH1	140	220	175	2000	0.0128	1.50	0.93	12.0	82	hooked	0.5	80	1100	119	S
2017	SH2	140	220	175	2000	0.0128	1.50	0.93	12.0	83.2	hooked	1	80	1100	156	S
[69]	SH3	140	220	175	2000	0.0128	1.50	0.93	12.0	83.8	hooked	1.5	80	1100	187	S
	SH4	140	220	175	2000	0.0128	2.50	1.93	12.0	82	hooked	0.5	80	1100	63	S
	SH5	140	220	175	2000	0.0128	2.50	1.93	12.0	83.2	hooked	1	80	1100	80	S
	SH6	140	220	175	2000	0.0128	2.50	1.93	12.0	83.8	hooked	1.5	80	1100	136	S
Arslan et al.	A2.5F1.0A	150	230	200	1000	0.0134	2.50	2.00	22.0	33.68	hooked	1	55	1100	65	S
2017	A2.5F1.0b	150	230	200	1000	0.0134	2.50	2.00	22.0	24.53	hooked	1	55	1100	44	S
[70]	A2.5F2.0	150	230	200	1000	0.0134	2.50	2.00	22.0	21.43	hooked	2	55	1100	50	S
	A2.5F3.0	150	230	200	1000	0.0134	2.50	2.00	12.0	9.77	hooked	3	55	1100	39	S
	A3.5F1.0	150	230	200	1400	0.0134	3.50	3.00	22.0	20.21	hooked	1	55	1100	33	S
	A3.5F2.0	150	230	200	1400	0.0134	3.50	3.00	22.0	21.43	hooked	2	55	1100	43	S
	A3.5F3.0	150	230	200	1400	0.0134	3.50	3.00	12.0	27.91	hooked	3	55	1100	59	S
	A4.5F1.0	150	230	200	1800	0.0134	4.50	4.00	22.0	24.53	hooked	1	55	1100	43	S
	A4.5F2.0	150	230	200	1800	0.0134	4.50	4.00	22.0	21.43	hooked	2	55	1100	36	S-FL
Parra-Montesinos et al.	11	152	457.2	381	2766.2	0.0271	3.40	3.41	10.0	49.2	hooked	1	80	1100	174	NA
2006	7	152	457.2	381	2766.2	0.0271	3.40	3.41	10.0	31	hooked	1.5	60	1100	151	NA
[71]	10	152	457.2	381	2766.2	0.0271	3.40	3.41	10.0	44.9	hooked	1.5	60	1100	191	NA
	9	152	457.2	381	2766.2	0.0271	3.40	3.41	10.0	44.9	hooked	1.5	60	1100	192	NA
	12	152	457.2	381	2766.2	0.0271	3.40	3.41	10.0	49.2	hooked	1	80	1100	220	NA
	8	152	457.2	381	2766.2	0.0271	3.40	3.41	10.0	31	hooked	1.5	60	1100	198	NA
	4	152	457.2	381	2817	0.0271	3.50	3.47	10.0	38.1	hooked	1	60	1100	149	NA
	3	152	457.2	381	2817	0.0271	3.50	3.47	10.0	38.1	hooked	1	60	1100	203	NA
	1	152	457.2	381	2817	0.0197	3.50	3.47	10.0	38.1	hooked	1	60	1100	178	NA
	2	152	457.2	381	2817	0.0197	3.50	3.47	10.0	38.1	hooked	1	60	1100	181	NA
Rosenbusch & Teutsch	2.2/2	200	300	260	952.64	0.0181	1.50	1.51	10.0	41.2	hooked	0.25	67	1100	280	NA
2003	2.2/3	200	300	260	952.64	0.0181	1.50	1.51	10.0	40.3	hooked	0.76	67	1100	300	NA
[72]	2.4/2	200	300	260	1450.48	0.0181	2.50	2.46	10.0	40	hooked	0.25	67	1100	108	NA
	2.4/3	200	300	260	1450.48	0.0181	2.50	2.46	10.0	38.7	hooked	0.76	67	1100	144	NA
	2.3/2	200	300	260	1450.48	0.0115	2.50	2.46	10.0	40	hooked	0.25	67	1100	82	NA
	2.3/3	200	300	260	1450.48	0.0115	2.50	2.46	10.0	38.7	hooked	0.76	67	1100	107	NA
	T15*100-SFRC-2	200	500	460	3248.8	0.0280	3.40	3.35	10.0	37.7	hooked	0.5	67	1100	244	NA
	T23*50-SFRC-2	200	500	460	3248.8	0.0280	3.40	3.35	10.0	38.8	hooked	0.5	67	1100	252	NA
	T15*75-SFRC-2	200	500	460	3248.8	0.0280	3.40	3.35	10.0	37.7	hooked	0.5	67	1100	259	NA
	T15*50-SFRC-1	200	500	460	3248.8	0.0280	3.40	3.35	10.0	37.7	hooked	0.5	67	1100	263	NA
	1.2/2	200	300	260	1948.32	0.0356	3.50	3.42	10.0	46.9	hooked	0.25	67	1100	110	NA
	1.2/3	200	300	260	1948.32	0.0356	3.50	3.42	10.0	43.7	hooked	0.51	67	1100	120	NA
	1.2/4	200	300	260	1948.32	0.0356	3.50	3.42	10.0	48.3	hooked	0.76	67	1100	155	NA
	20*30-SFRC-1	200	300	260	1968.64	0.0283	3.50	3.46	10.0	37.7	hooked	0.5	67	1100	111	NA
	20*30-SFRC-2	200	300	260	1968.64	0.0283	3.50	3.46	10.0	38.8	hooked	0.5	67	1100	132	NA
	20*60-SFRC-1	200	600	540	3929.52	0.0273	3.50	3.48	10.0	37.7	hooked	0.25	67	1100	153	NA
	20*60-SFRC-2	200	600	560	3929.52	0.0273	3.50	3.36	10.0	38.8	hooked	0.5	67	1100	230	NA
	2.6/2	200	300	260	2253.12	0.0181	4.00	4.01	10.0	41.2	hooked	0.25	67	1100	82	NA
	2.6/3	200	300	260	2253.12	0.0181	4.00	4.01	10.0	40.3	hooked	0.76	67	1100	117	NA
Sahoo et al.	TB0.75_1.6	150	250	217	1150	0.0185	1.59	1.13	10.0	35	hooked	0.75	80	1100	149	DT
2016	TB0.75_2.5	150	250	217	1600	0.0185	2.47	2.00	10.0	35	hooked	0.75	80	1100	99	SC
[73]	TB0.75_3.0	150	250	217	1750	0.0185	2.95	2.49	10.0	35	hooked	0.75	80	1100	85	SC
Amin & Foster	B25-0-0-	300	700	622	4500	0.0198	2.81	2.49	10.0	34	hooked	0.321	65	2300	286	S
2016 [74]	B50-0-0	300	700	622	4500	0.0198	2.81	2.49	10	36	hooked	0.687	65	2300	356	S
Tahenni et al.	S0F0.5-65	100	150	135	900	0.0116	2.22	2.15	15.0	64.2	hooked	0.5	65	1100	42	S
2016	S0F0.5-65	100	150	135	900	0.0116	2.22	2.15	15.0	64.2	hooked	0.5	65	1100	44	S
[75]	S0F0.5-65	100	150	135	900	0.0116	2.22	2.15	15.0	64.2	hooked	0.5	65	1100	43	S
	S0F1.0-65	100	150	135	900	0.0116	2.22	2.15	15.0	64	hooked	1	65	1100	45	S-FL
	S0F1.0-65	100	150	135	900	0.0116	2.22	2.15	15.0	64	hooked	1	65	1100	48	S-FL
	S0F1.0-65	100	150	135	900	0.0116	2.22	2.15	15.0	64	hooked	1	65	1100	43	S-FL
	S0F1.0-80	100	150	135	900	0.0116	2.22	2.15	15.0	60	hooked	1	80	1100	50	S-FL
	S0F1.0-80	100	150	135	900	0.0116	2.22	2.15	15.0	60	hooked	1	80	1100	52	S-FL
	S0F1.0-80	100	150	135	900	0.0116	2.22	2.15	15.0	60	hooked	1	80	1100	45	S-FL
Narayanan & Darwish	SF1	85	150	130	900	0.0205	2.02	1.94	9.6	51.85	crimped	0.25	100	2000	33	S
1987	SF2	85	150	130	1030	0.0205	2.52	2.44	9.6	51.85	crimped	0.25	100	2000	30	S
[76]	SF3	85	150	130	1160	0.0205	3.02	2.94	9.6	51.85	crimped	0.25	100	2000	31	S
	SF4	85	150	130	900	0.0205	2.02	1.94	9.6	33.32	crimped	0.25	100	2000	30	S
	SF5	85	150	130	1030	0.0205	2.52	2.44	9.6	33.32	crimped	0.25	100	2000	23	S
	SF6	85	150	130	1160	0.0205	3.02	2.94	9.6	33.32	crimped	0.25	100	2000	22	S
	B1	85	150	130	1160	0.0205	3.02	2.94	9.6	51.68	crimped	0.5	133	2000	36	S
	B7	85	150	130	1160	0.0205	3.02	2.94	9.6	30.6	crimped	0.5	133	2000	22	S
	B9	85	150	130	1160	0.0205	3.02	2.94	9.6	31.025	crimped	1	100	2000	33	S
	B11	85	150	130	900	0.0205	2.02	1.94	9.6	51.68	crimped	0.5	133	2000	51	S
	B12	85	150	130	1030	0.0205	2.52	2.44	9.6	51.68	crimped	0.5	133	2000	41	S
	B13	85	150	130	1290	0.0205	3.52	3.44	9.6	41.65	crimped	0.5	133	2000	29	S
	B14	85	150	130	900	0.0205	2.02	1.94	9.6	48.705	crimped	1	133	2000	62	S
	B15	85	150	130	1030	0.0205	2.52	2.44	9.6	48.705	crimped	1	133	2000	49	S
	B16	85	150	130	1290	0.0205	3.52	3.44	9.6	48.79	crimped	1	133	2000	33	S
	B17	85	150	128	1160	0.0370	3.06	2.98	9.6	41.65	crimped	0.5	133	2000	32	S
	B18	85	150	126	1160	0.0572	3.11	3.03	9.6	41.65	crimped	0.5	133	2000	38	S
	B19	85	150	128	1160	0.0370	3.06	2.98	9.6	30.6	crimped	0.5	133	2000	25	S
	B20	85	150	126	1160	0.0572	3.11	3.03	9.6	30.6	crimped	0.5	133	2000	25	S
	B23	85	150	128	1160	0.0370	3.06	2.98	9.6	48.79	crimped	1	133	2000	48	S
	B24	85	150	126	1160	0.0572	3.11	3.03	9.6	48.79	crimped	1	133	2000	54	S
	B25	85	150	126	1160	0.0572	3.11	3.03	9.6	53.55	crimped	1.5	100	2000	52	S
	B26	85	150	126	1160	0.0572	3.11	3.03	9.6	43.18	crimped	2	100	2000	53	S
	B27	85	150	128	1160	0.0370	3.06	2.98	9.6	53.55	crimped	1.5	100	2000	49	S
	B28	85	150	126	900	0.0572	2.08	2.00	9.6	50.15	crimped	0.5	100	2000	59	S
	B29	85	150	126	900	0.0572	2.08	2.00	9.6	45.9	crimped	1	100	2000	73	S
	B30	85	150	126	900	0.0572	2.08	2.00	9.6	53.55	crimped	1.5	100	2000	77	S
	B31	85	150	126	900	0.0572	2.08	2.00	9.6	43.18	crimped	2	100	2000	68	S
Cucchiara et al.	A10	150	250	219	2300	0.0191	2.80	2.75	10.0	40.85	hooked	1	60	1115	97	S
2004	A20	150	250	219	2300	0.0191	2.80	2.75	10.0	40.85	hooked	2	60	1115	104	S
[77]	B10	150	250	219	2300	0.0191	2.00	1.95	10.0	43.23	hooked	1	60	1115	116	S
	B20	150	250	219	2300	0.0191	2.00	1.95	10.0	43.23	hooked	2	60	1115	117	S
Kwak et al.	FHB2-2	125	250	212	1248	0.0152	2.00	1.46	19.0	63.8	hooked	0.5	63	1079	135	S-FL
2002	FHB3-2	125	250	212	1248	0.0152	2.00	1.46	19.0	68.6	hooked	0.75	63	1079	145	S-FL
[48]	FNB2-2	125	250	212	1248	0.0152	2.00	1.46	19.0	30.8	hooked	0.5	63	1079	108	S
	FNB2-3	125	250	212	1672	0.0152	3.00	2.46	19.0	30.8	hooked	0.5	63	1079	68	S
Lim & Oh	S0.00V1	100	180	130	1300	0.0309	3.08	3.00	10.0	38.69	straight smooth	1	60	1303	59	S
1999 [78]	S0.00V2	100	180	130	1300	0.0309	3.08	3.00	10.0	42.4	straight smooth	2	60	1303	75	S
Dinh et al.	B18-1a	152	455	381	2136	0.0196	3.44	3.04	10.0	44.8	hooked	0.75	55	1100	172	SC + DT + Y
2010	B18-1b	152	455	381	2136	0.0196	3.44	3.04	10.0	44.8	hooked	0.75	55	1100	162	SC + DT + Y
[79]	B18-2a	152	455	381	2136	0.0196	3.44	3.04	10.0	38.1	hooked	1	55	1100	171	SC + DT + Y
	B18-2b	152	455	381	2136	0.0196	3.44	3.04	10.0	38.1	hooked	1	55	1100	174	SC + DT + Y
	B18-3a	152	455	381	2136	0.0263	3.44	3.04	10.0	31	hooked	1.5	55	1100	150	ST + DT + B
	B18-3b	152	455	381	2136	0.0263	3.44	3.04	10.0	31	hooked	1.5	55	1100	198	SC + ST
	B18-3c	152	455	381	2136	0.0263	3.44	3.04	10.0	44.9	hooked	1.5	55	1100	193	ST + DT
	B18-3d	152	455	381	2136	0.0263	3.44	3.04	10.0	44.9	hooked	1.5	55	1100	191	ST + DT
	B18-5a	152	455	381	2136	0.0263	3.44	3.04	10.0	49.2	hooked	1	80	1100	174	DT
	B18-5b	152	455	381	2136	0.0263	3.44	3.04	10.0	49.2	hooked	1	80	1100	220	ST + DT
	B18-7a	152	455	381	2136	0.0196	3.44	3.04	10.0	43.3	hooked	0.75	80	2300	194	ST + DT + Y
	B18-7b	152	455	381	2136	0.0196	3.44	3.04	10.0	43.3	hooked	0.75	80	2300	191	ST + DT + Y
	B27-1a	205	685	610	3558	0.0196	3.50	3.21	10.0	50.8	hooked	0.75	55	1100	369	ST + DT
	B27-1b	205	685	610	3558	0.0196	3.50	3.21	10.0	50.8	hooked	0.75	55	1100	341	DT
	B27-2a	205	685	610	3558	0.0196	3.50	3.21	10.0	28.7	hooked	0.75	80	1100	355	SC + ST
	B27-2b	205	685	610	3558	0.0196	3.50	3.21	10.0	28.7	hooked	0.75	80	1100	348	DT
	B27-3b	205	685	610	3558	0.0152	3.50	3.21	10.0	42.3	hooked	0.75	55	1100	351	SC + ST + Y
	B27-4a	205	685	610	3558	0.0152	3.50	3.21	10.0	29.6	hooked	0.75	80	1100	271	ST + DT + B
	B27-4b	205	685	610	3558	0.0152	3.50	3.21	10.0	29.6	hooked	0.75	80	1100	228	ST + DT + B
	B27-5	205	685	610	3558	0.0196	3.50	3.21	10.0	44.4	hooked	1.5	55	1100	438	SC + ST + Y
	B27-6	205	685	610	3558	0.0196	3.50	3.21	10.0	42.8	hooked	1.5	80	1100	424	ST + DT + Y
Lima Araujo et al.	V-1-0	150	390	340	2200	0.0308	2.50	2.21	12.5	58.87	hooked	1	65	1150	262	NA
2014 [80]	V-2-0	150	390	340	2200	0.0308	2.50	2.21	12.5	51.67	hooked	2	65	1150	292	NA
Casanova et al.	FRC1	150	800	735	5600	0.0106	3.81	3.67	12.5	42	hooked	1.25	75	1200	360	NA
1997	FRC2	150	800	735	5600	0.0106	3.81	3.67	12.5	38	hooked	1.25	60	1200	360	NA
[81]	HSFRC1	125	250	225	2000	0.0349	2.89	2.44	10.0	90	hooked	1.25	60	1200	158	DT
Aoude et al.	A0.5%	150	250	202	1700	0.0117	2.97	2.48	10.0	21.3	hooked	0.5	55	1100	48	S
2012	A1%	150	250	202	1700	0.0117	2.97	2.48	10.0	19.6	hooked	1	55	1100	57	S
[82]	B0.5%	300	500	437	3700	0.0150	3.09	2.86	10.0	21.3	hooked	0.5	55	1100	161	S
	B1%	300	500	437	3700	0.0150	3.09	2.86	10	19.6	hooked	1	55	1100	205	S
Minelli & Plizzari	NSC1-FRC1	200	480	435	4350	0.0104	2.51	2.30	20	24.8	hooked	0.38	50	1100	134	S
2013	NSC2-FRC1	200	480	435	4350	0.0104	2.51	2.30	20	33.5	hooked	0.38	50	1100	120	S
[83]	NSC2-FRC2	200	480	435	4350	0.0104	2.51	2.30	20	33.5	hooked + straight	0.57	78	1333	142	S
	NSC3-FRC	200	480	435	4350	0.0104	2.51	2.30	20	38.6	hooked	0.38	50	1100	141	S
	HSC1-FRC1	200	480	435	4350	0.0104	2.51	2.30	20	61.1	hooked	0.64	48	1250	191	S-FL
	NSC4-FRC-500-1	200	500	455	2280	0.0099	2.51	2.31	15	24.4	hooked	0.25	50	1100	197	S-FL
	NSC4-FRC-500-2	200	500	455	2280	0.0099	2.51	2.31	15	24.4	hooked	0.25	50	1100	157	S
	NSC4-FRC-1000	200	1000	910	4550	0.0104	2.50	2.40	20	24.4	hooked	0.25	50	1100	258	S
	HSC2-FRC-1000	200	1000	910	4550	0.0104	2.50	2.40	20	55	hooked	0.25	50	1100	339	S
Kang et al.	FLB-0.5-2	125	250	210	1250	0.0153	2.00	1.52	19	44.6	hooked	0.5	63	1100	82	S-FL
2011	FLB-0.5-4	125	250	210	2100	0.0153	4.00	3.52	19	44.6	hooked	0.5	63	1100	36	S
[84]	FNB-0.5-2	125	250	210	1250	0.0153	2.00	1.52	19	57.2	hooked	0.5	63	1100	78	S-FL
Casanova & Rossi	HSFRC1	125	250	225	2000	0.0349	2.89	2.44	10	90	hooked	1.25	60	1200	139	S
1999 [85]	HSFRC2	125	250	225	2000	0.0349	2.89	2.44	10	90	hooked	1.25	60	1200	139	S
Lim et al.	2/0.5/2.5	152	254	221	2100	0.0120	2.50	2.45	10	34	hooked	0.5	60	1130	59	S
1987	4/1.0/1.5	152	254	221	1600	0.0239	1.50	1.45	10	34	hooked	1	60	1130	148	S
[44]	4/1.0/2.5	152	254	221	2100	0.0239	2.50	2.45	10	34	hooked	1	60	1130	84	S
	4/1.0/3.5	152	254	221	2100	0.0239	3.50	3.45	10	34	hooked	1	60	1130	68	S-FL
	4/0.5/1.5	152	254	221	1600	0.0239	1.50	1.45	10	34	hooked	0.5	60	1130	136	S
	4/0.5/2.5	152	254	221	2100	0.0239	2.50	2.45	10	34	hooked	0.5	60	1130	65	S
	4/0.5/3.5	152	254	221	2100	0.0239	3.50	3.45	10	34	hooked	0.5	60	1130	50	S
Mansur et al.	B1	150	225	197	1288	0.0136	2.00	1.49	20	29.1	hooked	0.5	60	1260	76	SC
1986	B2	150	225	197	1603.2	0.0136	2.80	2.29	20	29.1	hooked	0.5	60	1260	53	SC
[54]	B3	150	225	197	1918.4	0.0136	3.60	3.09	20	29.1	hooked	0.5	60	1260	46	DT
	C1	150	225	197	1288	0.0136	2.00	1.49	20	29.9	hooked	0.75	60	1260	86	SC
	C2	150	225	197	1603.2	0.0136	2.80	2.29	20	29.9	hooked	0.75	60	1260	61	SC
	C6	150	225	197	1603.2	0.0204	2.80	2.29	20	29.9	hooked	0.75	60	1260	66	SC
	E2	150	225	197	1603.2	0.0136	2.80	2.29	20	20.6	hooked	0.75	60	1260	46	SC
	E3	150	225	197	1603.2	0.0204	2.80	2.29	20	20.6	hooked	0.75	60	1260	61	SC
	F3	150	225	197	1603.2	0.0204	2.80	2.29	20	33.4	hooked	0.75	60	1260	87	SC
Zarrinpour & Chao	SFRC12W6	152	305	254	1778	0.0248	3.50	2.90	10	29	hooked	0.75	67	1096	121	S
2017	SFRC12W24	610	305	254	1778	0.0247	3.50	2.90	10	29	hooked	0.75	67	1096	482	S
[86]	SFRC18a	152	457	394	2844.8	0.0286	3.61	3.22	10	39	hooked	0.75	67	1096	163	S
	SFRC18b	152	457	394	2844.8	0.0286	3.61	3.22	10	39	hooked	0.75	67	1096	196	S
	SFRC24a	203	610	541	3733.8	0.0254	3.45	3.17	10	50	hooked	0.75	67	1096	273	S
	SFRC24b	203	610	541	3733.8	0.0254	3.45	3.17	10	50	hooked	0.75	67	1096	386	S
	SFRC36a	254	915	813	5689.6	0.0270	3.50	3.31	10	50	hooked	0.75	67	1096	700	S
	SFRC36b	254	915	813	5689.6	0.0270	3.50	3.31	10	50	hooked	0.75	67	1096	721	S
	SFRC48a	305	1220	1118	7823.2	0.0255	3.50	3.35	10	50	hooked	0.75	67	1096	1081	S
	SFRC48b	305	1220	1118	7823.2	0.0255	3.50	3.35	10	50	hooked	0.75	67	1096	1044	S
Noghabai	HSC.I.S6.0.15	200	250	180	1200	0.0447	3.33	2.22	16	90.6	straight smooth	1	40	2600	300	S
2000	HSC.I.Smix	200	250	180	1200	0.0447	3.33	2.22	16	83.2	hooked + straight	1	48	1850	296	S
[87]	HSC.I.S60/0.7/0.5	200	250	180	1200	0.0447	3.33	2.22	16	80.5	hooked	0.5	86	2200	253	S
	HSC.I.S60/0.7/0.75	200	250	180	1200	0.0447	3.33	2.22	16	80.5	hooked	0.75	86	2200	263	S
	NSC.II.Smix	200	250	195	1200	0.0309	3.08	2.05	16	39.4	hooked + straight	1	48	1850	190	S
	HSC.II.S30/0.6	200	300	235	1300	0.0428	2.77	1.91	16	91.4	hooked	1	50	1100	311	S
	HSC.II.S6/0.15	200	300	235	1300	0.0428	2.77	1.91	16	93.3	straight smooth	1	40	2600	364	S
	HSC.II.Smix	200	300	235	1300	0.0428	2.77	1.91	16	89.6	hooked + straight	1	48	1850	408	S
	HSC.III.S6/0.15	200	500	410	3000	0.0306	2.93	2.44	18	76.8	straight smooth	1	40	2600	293	S
	HSC.III.S6/0.15	200	500	410	3000	0.0306	2.93	2.44	18	76.8	straight smooth	1	40	2600	340	S
	HSC.III.Smix	200	500	410	3000	0.0306	2.93	2.44	18	72	hooked + straight	1	48	1850	371	S
	HSC.III.Smix	200	500	410	3000	0.0306	2.93	2.44	18	72	hooked + straight	1	48	1850	331	S
	HSC.III.S60/0.7/0.5	200	500	410	3000	0.0306	2.93	2.44	18	69.3	hooked	0.5	86	2200	268	S
	HSC.III.S60/0.7/0.5	200	500	410	3000	0.0306	2.93	2.44	18	69.3	hooked	0.5	86	2200	316	S
	HSC.III.S60/0.7/0.75	200	500	410	3000	0.0306	2.93	2.44	18	60.2	hooked	0.75	86	2200	343	S
	HSC.IV.S60/0.7/0.75	200	500	410	3000	0.0306	2.93	2.44	18	75.7	hooked	0.75	86	2200	296	S
	HSC.III.S6/0.15	300	700	570	5000	0.0287	2.98	2.63	18	76.8	straight smooth	1	40	2600	458	S
	HSC.IV.Smix	300	700	570	5000	0.0287	2.98	2.63	18	72	hooked + straight	1	48	1850	609	S
	HSC.III.S60/0.7/0.75	300	700	570	5000	0.0287	2.98	2.63	18	60.2	hooked	0.75	86	2200	522	S
Randl et al.	B19	200	350	314	3000	0.0350	3.50	3.18	0.4	132	straight smooth	2	75	2000	254	S
2017	B25	200	350	314	3000	0.0350	3.50	3.18	0.4	154	straight smooth	2	75	2000	321	S
[88]	B30	200	350	314	3000	0.0350	3.50	3.18	0.4	146	straight smooth	2	75	2000	360	S
	B20	200	350	314	3000	0.0350	3.50	3.18	0.4	133	straight smooth	1	75	2000	269	S
	B24	200	350	314	3000	0.0350	3.50	3.18	0.4	143	straight smooth	1	75	2000	202	S
	B29	200	350	314	3000	0.0350	3.50	3.18	0.4	153	straight smooth	1	75	2000	311	S
Ashour et al.	B-2-1.0-L	125	250	215	1360	0.0037	2.00	1.95	10	92	hooked	1	75	260	46	NA
1992	B-4-1.0-L	125	250	215	2220	0.0037	4.00	3.95	10	92.6	hooked	1	75	260	25	NA
[59]	B-6-1.0-L	125	250	215	3080	0.0037	6.00	5.95	10	93.7	hooked	1	75	260	16	NA
	B-1-0.5-A	125	250	215	930	0.0283	1.00	0.95	10	99	hooked	0.5	75	260	245	NA
	B-2-0.5-A	125	250	215	1360	0.0283	2.00	1.95	10	99.1	hooked	0.5	75	260	130	NA
	B-4-0.5-A	125	250	215	2220	0.0283	4.00	3.95	10	95.4	hooked	0.5	75	260	62	NA
	B-6-0.5-A	125	250	215	3080	0.0283	6.00	5.95	10	95.83	hooked	0.5	75	260	54	NA
	B-1-1.0-A	125	250	215	930	0.0283	1.00	0.95	10	95.3	hooked	1	75	260	343	NA
	B-2-1.0-A	125	250	215	1360	0.0283	2.00	1.95	10	95.3	hooked	1	75	260	163	NA
	B-4-1.0-A	125	250	215	2220	0.0283	4.00	3.95	10	97.53	hooked	1	75	260	86	NA
	B-6-1.0-A	125	250	215	3080	0.0283	6.00	5.95	10	100.5	hooked	1	75	260	54	NA
	B-1-1.5-A	125	250	215	930	0.0283	1.00	0.95	10	96.4	hooked	1.5	75	260	375	NA
	B-2-1.5-A	125	250	215	1360	0.0283	2.00	1.95	10	96.6	hooked	1.5	75	260	194	NA
	B-4-1.5-A	125	250	215	2220	0.0283	4.00	3.95	10	97.1	hooked	1.5	75	260	95	NA
	B-6-1.5-A	125	250	215	3080	0.0283	6.00	5.95	10	101.32	hooked	1.5	75	260	54	NA
	B-2-1.0-M	125	250	215	1360	0.0458	2.00	1.95	10	94.5	hooked	1	75	260	181	NA
	B-4-1.0-M	125	250	215	2220	0.0458	4.00	3.95	10	93.8	hooked	1	75	260	105	NA
	B-6-1.0-M	125	250	215	3080	0.0458	6.00	5.95	10	95	hooked	1	75	260	80	NA
Tan et al.	2	140	375	340	1910	0.0167	2.00	1.71	19	35	hooked	0.5	60	1100	219	NA
1993	3	140	375	340	1910	0.0167	2.00	1.71	19	33	hooked	0.75	60	1100	182	NA
[89]	4	140	375	340	1910	0.0167	2.00	1.71	19	36	hooked	1	60	1100	212	NA
	5	140	375	340	1910	0.0167	2.50	2.21	19	36	hooked	1	60	1100	155	NA
	6	140	375	340	1910	0.0167	1.50	1.21	19	36	hooked	1	60	1100	308	NA
Pansuk et al.	NS08	150	400	350	2000	0.0561	2.86	2.57	2	121.1058	hooked	0.8	65	2000	342	NA
2017 [90]	NS16	150	400	350	2000	0.0561	2.86	2.57	2	120.3022	hooked	1.6	65	2000	533	NA
Kim et al.	21FB	260	400	340	3120	0.0172	4.00	3.71	10	21	hooked	0.75	60	1336	118	NA
2017 [91]	60FB	260	400	340	3120	0.0172	4.00	3.71	10	56	hooked	0.75	60	1336	208	NA
Sharma, 1986 [52]	S3F	150	300	276	1600	0.0146	1.81	1.78	10	48.6	hooked	0.96	85	1100	124	NA
Narayanan & Darwish	D2	100	400	345	1000	0.0355	0.70	0.43	5	52.89	crimped	0.25	100	2000	351	NA
1988	D3	100	400	345	1000	0.0355	0.70	0.43	5	51.004	crimped	0.5	100	2000	326	NA
[92]	D4	100	400	345	1000	0.0355	0.70	0.43	5	47.56	crimped	0.75	100	2000	362	NA
	D5	100	400	345	1000	0.0355	0.70	0.43	5	55.924	crimped	1	100	2000	397	NA
	D6	100	400	345	1000	0.0355	0.70	0.43	5	54.94	crimped	1.25	100	2000	394	NA
	D7	100	400	345	1000	0.0355	0.46	0.20	5	50.512	crimped	1	100	2000	455	NA
	D8	100	400	345	1000	0.0355	0.58	0.32	5	47.806	crimped	1	100	2000	405	NA
	D9	100	400	345	1000	0.0355	0.81	0.55	5	45.592	crimped	1	100	2000	343	NA
	D10	100	400	345	1000	0.0355	0.93	0.67	5	49.118	crimped	1	100	2000	345	NA
	D11	100	400	345	1000	0.0355	0.70	0.43	5	30.996	crimped	1	100	2000	295	NA
	D12	100	400	345	1000	0.0355	0.70	0.43	5	34.686	crimped	1	100	2000	334	NA
Li, Ward & Hamza	M1	63.5	127	102	612	0.0220	3.00	2.88	2.36	53	crimped	1	29	1000	17	NA
1992	M2	127	228	204	1224	0.0221	3.00	2.88	2.36	53	crimped	1	29	1000	51	NA
[93]	M3	63.5	127	102	612	0.0220	3.00	2.88	2.36	50.2	crimped	2	29	1000	21	NA
	M4	127	228	204	1224	0.0221	3.00	2.88	2.36	50.2	crimped	2	29	1000	67	NA
	M5	63.5	127	102	612	0.0220	3.00	2.88	2.36	62.6	crimped	1	29	1000	18	NA
	M6	127	228	204	1224	0.0221	3.00	2.88	2.36	62.6	crimped	1	29	1000	62	NA
	M8	63.5	127	102	204	0.0220	1.00	0.88	2.36	62.6	crimped	1	29	1000	51	NA
	M9	63.5	127	102	306	0.0220	1.50	1.38	2.36	62.6	crimped	1	29	1000	33	NA
	M10	63.5	127	102	357	0.0220	1.75	1.63	2.36	62.6	crimped	1	29	1000	30	NA
	M11	63.5	127	102	408	0.0220	2.00	1.88	2.36	62.6	crimped	1	29	1000	26	NA
	M12	63.5	127	102	459	0.0220	2.25	2.13	2.36	62.6	crimped	1	29	1000	23	NA
	M13	63.5	127	102	510	0.0220	2.50	2.38	2.36	62.6	crimped	1	29	1000	21	NA
	M14	63.5	127	102	561	0.0220	2.75	2.63	2.36	62.6	crimped	1	29	1000	18	NA
	M15	63.5	127	102	612	0.0110	3.00	2.88	2.36	62.6	crimped	1	29	1000	13	NA
	M16	63.5	127	102	612	0.0330	3.00	2.88	2.36	62.6	crimped	1	29	1000	18	NA
	M17	63.5	127	102	612	0.0330	3.00	2.88	2.36	54.1	crimped	1	57	1000	25	NA
	C1	127	228	204	1224	0.0221	3.00	2.88	9	22.7	hooked	1	60	1172	79	NA
	C2	63.5	127	102	612	0.0220	3.00	2.88	9	22.7	hooked	1	60	1172	21	NA
	C3	63.5	127	102	612	0.0110	3.00	2.88	9	22.7	hooked	1	60	1172	16	NA
	C4	63.5	127	102	306	0.0110	1.50	1.38	9	22.7	hooked	1	60	1172	37	NA
	C5	127	228	204	1224	0.0221	3.00	2.88	9	26	hooked	1	100	1172	79	NA
	C6	63.5	127	102	612	0.0220	3.00	2.88	9	26	hooked	1	100	1172	23	NA
Swamy et al.	1TLF-1	55	300	265	3000	0.0431	2.00	1.62	14	36.49	crimped	1	100	1570	81	S
1993	1TLF-2	55	300	265	3000	0.0431	3.43	3.05	14	41.902	crimped	1	100	1570	59	S
[94]	1TLF-3	55	300	265	3000	0.0431	4.91	4.53	14	36.9	crimped	1	100	1570	43	S
	2TLF-1	55	300	265	3000	0.0276	2.00	1.62	14	38.704	crimped	1	100	1570	72	S
	2TLF-2	55	300	265	3000	0.0276	3.43	3.05	14	33.948	crimped	1	100	1570	46	S
	2TLF-3	55	300	265	3000	0.0276	4.91	4.53	14	36.818	crimped	1	100	1570	43	S-FL
	3TLF-1	55	300	265	3000	0.0155	2.00	1.62	14	36.572	crimped	1	100	1570	68	S-FL
Adebar et al.	FC2	150	610	560	1500	0.0214	1.63	1.34	14	54.1	hooked	0.75	60	1200	278	S
1997	FC3	150	610	560	1500	0.0214	1.63	1.34	14	49.9	hooked	1.5	60	1200	326	S
[127]	FC8	150	610	560	1500	0.0214	1.63	1.34	14	54.8	hooked	0.4	60	1200	206	S
	FC9	150	610	560	1500	0.0214	1.63	1.34	14	56.5	hooked	0.6	60	1200	234	S
	FC10	150	610	560	1500	0.0214	1.63	1.34	14	46.9	hooked	0.4	60	1200	249	S
	FC11	150	610	560	1500	0.0214	1.63	1.34	14	40.8	hooked	0.6	60	1200	239	S
Cho & Kim	F30-0.5-13	120	200	167.5	720	0.0132	1.43	1.25	13	25.7	hooked	0.5	60	1100	61	S
2003	F30-1.0-13	120	200	167.5	720	0.0132	1.43	1.25	13	25.3	hooked	1	60	1100	80	S-FL
[95]	F30-1.5-13	120	200	167.5	720	0.0132	1.43	1.25	13	23.9	hooked	1.5	60	1100	85	S-FL
	F50-0.5-13	120	200	167.5	720	0.0132	1.43	1.25	13	57.8	hooked	0.5	60	1100	95	S-FL
	F60-1.0-13	120	200	167.5	720	0.0132	1.43	1.25	13	61.5	hooked	1	60	1100	103	S-FL
	F70-0.5-19	120	200	167.5	720	0.0282	1.43	1.25	13	70.5	hooked	0.5	60	1100	179	S
	F70-1.0-19	120	200	167.5	720	0.0282	1.43	1.25	13	67.3	hooked	1	60	1100	170	S
	F70-1.5-19	120	200	167.5	720	0.0282	1.43	1.25	13	67.3	hooked	1.5	60	1100	187	S
	F80-0.5-16	120	200	167.5	720	0.0200	1.43	1.25	13	82.4	hooked	0.5	60	1100	158	S
	F80-1.0-16	120	200	167.5	720	0.0200	1.43	1.25	13	81.1	hooked	1	60	1100	163	S-FL
	F80-0.5-19	120	200	167.5	720	0.0282	1.43	1.25	13	86.1	hooked	0.5	60	1100	154	S
	F80-1.0-19	120	200	167.5	720	0.0282	1.43	1.25	13	89.4	hooked	1	60	1100	171	S-FL
Greenough & Nehdi	S-HE-50-0.5	200	300	265	2000	0.0178	3.02	2.64	10	47.9	hooked	0.5	50	1100	92	S
2008	S-HE-50-0.75	200	300	265	2000	0.0178	3.02	2.64	10	38	hooked	0.75	50	1100	107	S
[50]	S-HE-50-1.0	200	300	265	2000	0.0178	3.02	2.64	10	42.2	hooked	1	50	1100	150	S
	S-FE-50-0.5	200	300	265	2000	0.0178	3.02	2.64	10	45.4	flat end	0.5	50	1100	117	S
	S-FE-50-0.75	200	300	265	2000	0.0178	3.02	2.64	10	44.4	flat end	0.75	50	1100	146	S
	S-FE-50-1.0	200	300	265	2000	0.0178	3.02	2.64	10	40.3	flat end	1	50	1100	148	S
	S-FE-30-0.5	200	300	265	2000	0.0178	3.02	2.64	10	53.7	flat end	0.5	43	1100	108	S
	S-FE-30-0.75	200	300	265	2000	0.0178	3.02	2.64	10	46	flat end	0.75	43	1100	124	S
	S-FE-30-1.0	200	300	265	2000	0.0178	3.02	2.64	10	42.2	flat end	1	43	1100	153	S
Kang et al.	FNB-50-1	200	355	310	3560	0.0113	2.55	1.98	9.5	39.8	hooked	0.375	80	1100	134	S
2012 [96]	FNB-50-3	200	355	285	3560	0.0333	2.77	2.16	9.5	39.8	hooked	0.375	80	1100	223	S
Dupont & Vandewalle	2	200	300	260	1800	0.0355	3.46	3.08	14	46.4	hooked	0.25	65	1100	111	S
2003	3	200	300	260	1800	0.0355	3.46	3.08	14	43.2	hooked	0.5	65	1100	121	S
[97]	4	200	300	260	1800	0.0355	3.46	3.08	14	47.6	hooked	0.75	65	1100	156	S
	14	200	300	260	2300	0.0181	1.54	1.15	14	40.7	hooked	0.25	65	1100	282	S
	15	200	300	260	2300	0.0181	1.54	1.15	14	42.4	hooked	0.75	65	1100	302	S
	17	200	300	262	2300	0.0115	2.48	2.10	14	39.1	hooked	0.25	65	1100	84	S
	18	200	300	262	2300	0.0115	2.48	2.10	14	38.6	hooked	0.75	65	1100	110	S
	20	200	300	260	2300	0.0181	2.50	2.12	14	39.1	hooked	0.25	65	1100	110	S
	21	200	300	260	2300	0.0181	2.50	2.12	14	38.6	hooked	0.75	65	1100	146	S
	23	200	300	260	2300	0.0181	4.04	3.65	14	40.7	hooked	0.25	65	1100	84	S
	24	200	300	260	2300	0.0181	4.04	3.65	14	42.4	hooked	0.75	65	1100	119	S
	26	200	300	262	2300	0.0115	2.48	2.10	14	26.5	hooked	0.25	45	1100	102	S
	27	200	300	262	2300	0.0115	2.48	2.10	14	27.2	hooked	0.75	45	1100	122	S
	29	200	300	260	2300	0.0181	2.50	2.12	14	26.5	hooked	0.25	45	1100	102	S
	30	200	300	260	2300	0.0181	2.50	2.12	14	27.2	hooked	0.75	45	1100	122	S
	31	200	300	262	2300	0.0115	2.48	2.10	14	47.4	hooked	0.5	65	1100	132	S
	32	200	300	260	2300	0.0181	2.50	2.12	14	46.8	hooked	0.5	65	1100	159	S
	33	200	300	262	2300	0.0115	2.48	2.10	14	45.4	hooked	0.5	80	1100	149	S
	41	200	350	305	3250	0.0103	2.46	2.13	14	34.4	hooked	0.57	80	1100	165	S
	43	200	350	305	3250	0.0103	2.46	2.13	14	30.2	hooked	0.38	80	1100	165	S-FL
Swamy & Bahia	B52	175	250	210	2800	0.0401	4.50	4.26	10	36.408	crimped	0.4	100	1050	81	DT
1985	B53	175	250	210	2800	0.0401	4.50	4.26	10	38.376	crimped	0.8	100	1050	116	S + SC
[99]	B54	175	250	210	2800	0.0401	4.50	4.26	10	40.836	crimped	1.2	100	1050	117	S + SC
	B55	175	250	210	2800	0.0310	4.50	4.26	10	39.114	crimped	0.8	100	1050	120	SC + FL
	B59	175	250	210	2800	0.0401	4.50	4.26	10	38.54	crimped	0.8	100	1050	71	DT
Batson et al.	H1	101	152	127	1828.8	0.0309	4.80	4.01	2	33.22	flat	0.22	102	1100	28	S-FL
1972	H2	101	152	127	1828.8	0.0309	4.80	4.01	2	33.22	flat	0.22	102	1100	28	S-FL
[100]	H3	101	152	127	1828.8	0.0309	4.80	4.01	2	33.22	flat	0.22	102	1100	27	S-FL
	I1	101	152	127	1828.8	0.0309	4.80	4.01	2	33.22	crimped	0.22	46	1100	28	S-FL
	I2	101	152	127	1828.8	0.0309	4.80	4.01	2	33.22	crimped	0.22	46	1100	28	S-FL
	I3	101	152	127	1828.8	0.0309	4.80	4.01	2	33.22	crimped	0.22	46	1100	27	S-FL
	A2	101	152	127	1828.8	0.0309	4.80	4.01	2	33.22	round	0.22	102	1100	27	S-FL
	B3	101	152	127	1828.8	0.0309	4.40	3.61	2	33.22	round	0.22	102	1100	32	S
	C1	101	152	127	1828.8	0.0309	4.20	3.41	2	33.22	round	0.22	102	1100	32	S
	C2	101	152	127	1828.8	0.0309	4.20	3.41	2	33.22	round	0.22	102	1100	28	S
	C3	101	152	127	1828.8	0.0309	4.20	3.41	2	33.22	round	0.22	102	1100	25	S
	D2	101	152	127	1828.8	0.0309	4.30	3.51	2	33.22	round	0.22	102	1100	30	S
	D3	101	152	127	1828.8	0.0309	4.30	3.51	2	33.22	round	0.22	102	1100	28	S
	E3	101	152	127	1828.8	0.0309	4.20	3.41	2	40.21	round	0.44	102	1100	33	S-FL
	F1	101	152	127	1828.8	0.0309	4.00	3.21	2	40.21	round	0.44	102	1100	33	S
	F2	101	152	127	1828.8	0.0309	4.00	3.21	2	40.21	round	0.44	102	1100	31	S
	F3	101	152	127	1828.8	0.0309	4.00	3.21	2	40.21	round	0.44	102	1100	33	S
	G1	101	152	127	1828.8	0.0309	4.40	3.61	2	33.22	round	0.22	102	1100	29	S
	G3	101	152	127	1828.8	0.0309	4.40	3.61	2	33.22	round	0.22	102	1100	27	S
	L1	101	152	127	1828.8	0.0309	4.00	3.21	2	33.22	crimped	0.22	62	1100	30	S
	L2	101	152	127	1828.8	0.0309	4.00	3.21	2	33.22	crimped	0.22	62	1100	31	S
	L3	101	152	127	1828.8	0.0309	4.00	3.21	2	33.22	crimped	0.22	62	1100	33	S
	M1	101	152	127	1828.8	0.0309	4.60	3.81	2	33.22	crimped	0.22	62	1100	26	S
	M2	101	152	127	1828.8	0.0309	4.40	3.61	2	33.22	crimped	0.22	62	1100	27	S
	M3	101	152	127	1828.8	0.0309	4.40	3.61	2	33.22	crimped	0.22	62	1100	26	S
	N1	101	152	127	1828.8	0.0309	5.00	4.21	2	33.22	crimped	0.22	62	1100	25	S
	N2	101	152	127	1828.8	0.0309	4.80	4.01	2	33.22	crimped	0.22	62	1100	23	S
	O1	101	152	127	1828.8	0.0309	4.00	3.21	2	40.21	crimped	0.44	62	1100	32	S
	P1	101	152	127	1828.8	0.0309	4.20	3.41	2	40.21	crimped	0.44	62	1100	34	S
	P2	101	152	127	1828.8	0.0309	4.20	3.41	2	40.21	crimped	0.44	62	1100	30	S
	P3	101	152	127	1828.8	0.0309	4.20	3.41	2	40.21	crimped	0.44	62	1100	33	S
	R1	101	152	127	1828.8	0.0309	3.20	2.41	2	39.72	crimped	0.88	62	1100	37	S
	R2	101	152	127	1828.8	0.0309	3.40	2.61	2	39.72	crimped	0.88	62	1100	35	S
	S1	101	152	127	1828.8	0.0309	3.40	2.61	2	39.72	crimped	0.88	62	1100	33	S
	S2	101	152	127	1828.8	0.0309	3.40	2.61	2	39.72	crimped	0.88	62	1100	42	S
	S3	101	152	127	1828.8	0.0309	3.40	2.61	2	39.72	crimped	0.88	62	1100	40	S
	U1	101	152	127	1828.8	0.0309	2.80	2.01	2	39.79	crimped	1.76	62	1100	57	S-FL
	V2	101	152	127	1828.8	0.0309	1.80	1.01	2	39.79	crimped	1.76	62	1100	77	S
	W1	101	152	127	1828.8	0.0309	1.20	0.41	2	39.79	crimped	1.76	62	1100	145	S
	W2	101	152	127	1828.8	0.0309	1.20	0.41	2	39.79	crimped	1.76	62	1100	140	S
	X1	101	152	127	1828.8	0.0309	4.80	4.01	2	33.22	crimped	0.22	62	1100	25	S
	X2	101	152	127	1828.8	0.0309	4.80	4.01	2	33.22	crimped	0.22	62	1100	24	S
	X3	101	152	127	1828.8	0.0309	4.80	4.01	2	33.22	crimped	0.22	62	1100	26	S
Zhao et al.	S0005	150	300	259.5	2100	0.0252	2.00	1.61	20	34.45	mill-cut	0.5	35	700	114	S
2018	S0010	150	300	259.5	2100	0.0252	2.00	1.61	20	36.08	mill-cut	1	35	700	139	S
[101]	S0015	150	300	259.5	2100	0.0252	2.00	1.61	20	37.13	mill-cut	1.5	35	700	157	S
	S0020	150	300	259.5	2100	0.0252	2.00	1.61	20	35.26	mill-cut	2	35	700	150	S
Jindal	C1	100	152.6	127	1524	0.0199	3.60	3.52	2	20.68966	straight mild steel	1	25	4913	21	S
1984	G1	100	152.6	127	762	0.0199	2.00	1.92	2	20.68966	brass-coated high strength steel	1	100	2350	30	S
[102]	G2	100	152.6	127	762	0.0199	2.40	2.32	2	20.68966	brass-coated high strength steel	1	100	2350	30	S
	H1	100	152.6	127	762	0.0199	2.00	1.92	2	20.68966	brass-coated high strength steel	1	83	2350	40	S
	H3	100	152.6	127	1524	0.0199	3.60	3.52	2	20.68966	brass-coated high strength steel	1	83	2350	29	S
	H4	100	152.6	127	1524	0.0199	4.80	4.72	2	20.68966	brass-coated high strength steel	1	83	2350	25	S
	J1	100	152.6	127	762	0.0199	2.00	1.92	2	20.68966	brass-coated high strength steel	1	63	2350	33	S
Shin, Oh & Ghosh	2-0.5-0.5	100	200	175	700	0.0359	2.00	1.94	13	80	smooth straight	0.5	100	1856	120	S
1994	2-0.5-1	100	200	175	700	0.0359	2.00	1.94	13	80	smooth straight	1	100	1856	130	S
[103]	3-0.5-0.5	100	200	175	1050	0.0359	3.00	2.94	13	80	smooth straight	0.5	100	1856	56	S
	3-0.5-1	100	200	175	1050	0.0359	3.00	2.94	13	80	smooth straight	1	100	1856	72	S
	4.5-0.5-0.5	100	200	175	1575	0.0359	4.50	4.44	13	80	smooth straight	0.5	100	1856	49	S
	4.5-0.5-1	100	200	175	1575	0.0359	4.50	4.44	13	80	smooth straight	1	100	1856	61	S
Imam, Vandewalle & Mortelmans	B16	200	350	300	3250	0.0308	1.75	1.42	10	109.5	hooked	0.75	75	2000	531	S-FL
1994	B6	200	350	300	3250	0.0308	2.50	2.17	10	110	hooked	0.75	75	2000	287	SC
[104]	B7	200	350	300	3250	0.0308	3.50	3.17	10	111.5	hooked	0.75	75	2000	212	DT
	B12	200	350	300	3250	0.0308	4.50	4.17	10	110.8	hooked	0.75	75	2000	215	DT
Huang et al. 2005 [106]	PB14	150	300	255	2000	0.0493	1.96	1.57	20	55.842	chopped with butt ends	1	47	700	254	SC
Kwak, Suh & Hsu	IAS1	152.4	304.8	282.575	1524	0.0199	2.50	2.14	9.525	33.06897	hooked	1	100	1100	137	S
1991	IAS2	152.4	304.8	282.575	1524	0.0199	2.50	2.14	9.525	33.24138	hooked	1	100	1100	146	S
[107]	IBS1	152.4	304.8	282.575	1524	0.0199	2.50	2.14	9.525	33.03448	hooked	2	100	1100	134	S
	IBS2	152.4	304.8	282.575	1524	0.0199	2.50	2.14	9.525	34.37931	hooked	2	100	1100	139	S
Roberts & Ho	F3.0B1	50	200	170	820	0.0237	2.41	2.12	10	32.062	brass-coated high strength steel	3	100	1100	33	S-FL
1982	F4.5B1	50	200	170	820	0.0237	2.41	2.12	10	39.278	brass-coated high strength steel	4.5	100	1100	36	S-FL
[108]	F3.0B2	50	200	170	552	0.0237	1.62	1.33	10	32.062	brass-coated high strength steel	3	100	1100	51	S-FL
	F4.5B2	50	200	170	552	0.0237	1.62	1.33	10	39.278	brass-coated high strength steel	4.5	100	1100	54	S-FL
	F3.0B3	50	200	170	274	0.0237	0.81	0.51	10	32.062	brass-coated high strength steel	3	100	1100	81	S
	F4.5B3	50	200	170	274	0.0237	0.81	0.51	10	39.278	brass-coated high strength steel	4.5	100	1100	108	S-FL
Hwang et al.	S-35-0.5	100	200	165.5	1500	0.0343	3.02	2.96	10	39.4	hooked	0.5	60	1200	31	DT
2013	S-35-1.0	100	200	165.5	1500	0.0343	3.02	2.96	10	39.2	hooked	1	60	1200	52	DT
[42,109]	S-35-1.5	100	200	165.5	1500	0.0343	3.02	2.96	10	40	hooked	1.5	60	1200	54	DT
	S-35-2.0	100	200	165.5	1500	0.0343	3.02	2.96	10	35.5	hooked	2	60	1200	48	DT
	HS-50-1.0	100	200	159	1500	0.0478	3.14	3.08	10	58	hooked	1	60	1200	74	DT
	HS-70-00.5	100	200	159	1500	0.0478	3.14	3.08	10	80.1	hooked	0.5	60	1200	73	DT
	HS-70-1.0	100	200	159	1500	0.0478	3.14	3.08	10	88	hooked	1	60	1200	82	SC
Spinella et al.	A10	150	250	219	2300	0.0191	2.80	2.75	10	80.04	hooked	1	55	1100	115	S
2012 [110]	B10	150	250	219	2300	0.0191	2.00	1.95	10	80.04	hooked	1	55	1100	142	S
Chalioris & Sfiri, 2011 [111]	MF40	100	300	275	1450	0.0055	2.00	1.96	9.5	28.4	hooked	0.5	75	1100	43	S
Cohen & Aoude, 2012 [112]	M15-0.5%	125	250	212	2400	0.0152	3.77	3.07	10	59.4	hooked	0.5	55	1100	44	S
Aoude & Cohen	M15-0.5%H	125	250	212	2400	0.0152	3.77	3.07	10	49.6	hooked	0.5	80	1100	46	S
2014	M20-0.75%	125	250	210	2400	0.0228	3.81	3.10	10	49.7	hooked	0.75	55	1100	45	S
[113]	M20-1.0%	125	250	210	2400	0.0228	3.81	3.10	10	51.5	hooked	1	55	1100	59	S
	M20-1.0%A	125	250	210	2400	0.0228	3.81	3.10	12	54.5	hooked	1	55	1100	60	S
Qissab & Salman	G1B2	100	170	140	1100	0.0112	1.07	0.50	12.5	36.08	hooked	0.5	63	1100	73	S
2018	G1B3	100	170	140	1100	0.0112	1.07	0.50	12.5	36.9	hooked	0.75	63	1100	87	S
[114]	G1B5	100	170	140	1100	0.0112	2.50	1.93	12.5	36.08	hooked	0.5	63	1100	41	S
	G1B6	100	170	140	1100	0.0112	2.50	1.93	12.5	36.9	hooked	0.75	63	1100	51	S-FL
	G2B2	100	180	150	1100	0.0105	1.00	0.47	12.5	36.08	hooked	0.5	63	1100	107	S-FL
	G2B3	100	180	150	1100	0.0105	1.00	0.47	12.5	36.9	hooked	0.75	63	1100	126	S-FL
	G2B5	100	180	150	1100	0.0105	2.33	1.80	12.5	36.08	hooked	0.5	63	1100	45	S
	G2B6	100	180	150	1100	0.0105	2.33	1.80	12.5	36.9	hooked	0.75	63	1100	47	S
	G3B1	100	200	170	1100	0.0092	2.41	1.94	12.5	36.08	hooked	0.5	63	1100	42	S
	G3B2	100	200	170	1100	0.0092	1.29	0.82	12.5	36.08	hooked	0.5	63	1100	22	S
	G3B3	100	275	245	1100	0.0064	0.90	0.57	12.5	36.08	hooked	0.5	63	1100	51	S
Furlan & de Hanai	P3B	100	100	85.25	900	0.0166	3.52	3.40	10	54.8	crimped	1	127	1100	20	ST
1997	P4B	100	100	85.25	900	0.0166	3.52	3.40	10	50	crimped	2	127	1100	22	S-FL
[115]	P5A	100	100	85.25	900	0.0166	3.52	3.40	10	49.3	crimped	1	191	1100	22	ST
	P5B	100	100	85.25	900	0.0166	3.52	3.40	10	49.3	crimped	1	191	1100	19	ST
	P6B	100	100	85.25	900	0.0166	3.52	3.40	10	53.7	crimped	2	191	1100	20	ST
	P7A	100	100	85.25	900	0.0166	3.52	3.40	10	53.5	crimped	0.5	191	1100	23	ST
	P7B	100	100	85.25	900	0.0166	3.52	3.40	10	53.5	crimped	0.5	191	1100	18	ST
Dancygier & Savir	H3-S0-1_35	200	325	273	2000	0.0348	2.75	2.71	22	110.9	hooked	0.75	64	1000	202	S
2011 [116]	H3-S0-1_60	200	325	273	2000	0.0348	2.75	2.71	22	109.2	hooked	0.75	67	1000	211	S
Krassowska & Kosior-Kazberuk	A-IV-WS1.0	80	180	165	987	0.0171	2.99	2.83	4	41.23	hooked	1	50	800	33	S
2018 [117]	A-IV-WS1.5	80	180	165	987	0.0171	2.99	2.83	4	39.87	hooked	1.5	50	800	41	S
Yoo & Yang	S-F0.75	300	500	420	3700	0.0322	3.21	3.19	20	62.3	hooked	0.75	65	1400	417	S
2018	M-F0.75	450	750	648	5220	0.0327	3.26	3.24	20	62.3	hooked	0.75	65	1400	815	S
[118]	L-F0.75	600	1000	887	6780	0.0343	3.26	3.25	20	62.3	hooked	0.75	65	1400	1481	S
Gali & Subramaniam	SFRC_0.5_1	125	250	222	1200	0.0145	1.80	1.76	10	30	hooked	0.5	80	1225	79	S
2017 [119]	SFRC_0.5_2	125	250	222	1200	0.0145	1.80	1.76	10	30	hooked	0.5	80	1225	86	S
Zamanzadeh et al.	S_W70	70	300	270	1040	0.0332	2.56	2.52	10	50	recycled	0.769	58	1100	82	S
2015	S_W110	110	300	270	1040	0.0212	2.56	2.52	10	50	recycled	0.769	58	1100	96	S
[120]	S_W150	150	300	270	1040	0.0155	2.56	2.52	10	50	recycled	0.769	58	1100	110	S
Shoaib, Lubell & Bindiganavile	N31	310	308	258	1548	0.0250	3.00	2.42	10	23	hooked	1	55	1100	212	S
2014 [121]	N32	310	308	240	1440	0.0403	3.00	2.38	10	41	hooked	1	55	1100	282	S
Shoaib, 2012 [122]	H31	310	308	258	1548	0.0250	3.00	2.42	10	41	hooked	1	55	1100	279	S-FL
	H32	310	308	240	1440	0.0403	3.00	2.38	10	80	hooked	1	55	1100	459	S-FL
	N61	300	600	531	3186	0.0188	3.00	2.72	10	23	hooked	1	55	1100	255	S
	N62	300	600	523	3138	0.0255	3.00	2.71	10	23	hooked	1	55	1100	245	S
	H62	300	600	523	3138	0.0255	3.00	2.71	10	41	hooked	1	55	1100	447	S
	N10-1	300	1000	923	5538	0.0144	3.00	2.84	10	41	hooked	1	55	1100	500	S
	N10-2	300	1000	920	5520	0.0203	3.00	2.84	10	41	hooked	1	55	1100	505	S
	H10-1	300	1000	923	5538	0.0144	3.00	2.84	10	80	hooked	1	55	1100	653	S
	H10-2	300	1000	920	5520	0.0203	3.00	2.84	10	80	hooked	1	55	1100	651	S
Bae, Choi & Choi	U-0-f-3.5	200	350	300	2300	0.0360	3.50	3.17	2	215	hooked	2	55	1100	374	S
2014 [123]	U-0-f-2.0	200	350	300	1200	0.0360	2.00	1.67	2	199	hooked	2	55	1100	587	S
Abdul-Zaher et al.	B2	120	300	266	1100	0.0126	1.13	1.09	20	31.9	corrugated	0.2	50	834	127	S
2016	B3	120	300	266	1100	0.0126	1.13	1.09	20	31.9	corrugated	0.4	50	834	133	S
[124]	B4	120	300	266	1100	0.0126	1.13	1.09	20	31.9	corrugated	0.6	50	834	146	S
